# An RNAi-Based Suppressor Screen Identifies Interactors of the Myt1 Ortholog of *Caenorhabditis elegans*

**DOI:** 10.1534/g3.114.013649

**Published:** 2014-10-08

**Authors:** Anna K. Allen, Jessica E. Nesmith, Andy Golden

**Affiliations:** Laboratory of Biochemistry and Genetics, National Institute of Diabetes and Digestive and Kidney Diseases, National Institutes of Health, Bethesda, Maryland 20892

**Keywords:** WEE-1.3, fertility, suppressor, oocyte maturation, EGA

## Abstract

Oocyte maturation in all species is controlled by a protein complex termed the maturation promoting factor (MPF). MPF comprises a cyclin-dependent kinase (CDK) and its partner cyclin, and it is regulated by dueling regulatory phosphorylation events on the CDK. In *Caenorhabditis elegans*, the Wee1/Myt1 ortholog WEE-1.3 provides the inhibitory phosphorylations on CDK-1 that keep MPF inactive and halt meiosis. Prior work has shown that depletion of WEE-1.3 in *C. elegans* results in precocious oocyte maturation *in vivo* and a highly penetrant infertility phenotype. This study sought to further define the precocious maturation phenotype and to identify novel interactors with WEE-1.3. We found that WEE-1.3 is expressed throughout the germline and in developing embryos in a perinuclear pattern, and demonstrated that oocytes in WEE-1.3–depleted germlines have begun to transcribe embryonic genes and exhibit inappropriate expression of proteins normally restricted to fertilized eggs. In addition, we performed an RNAi suppressor screen of the infertile phenotype to identify novel factors that, when co-depleted with WEE-1.3, restore fertility to these animals. We screened ∼1900 essential genes by RNAi feeding and identified 44 (∼2% of the tested genes) that are suppressors of the WEE-1.3 depletion phenotype. The suppressors include many previously unidentified players in the meiotic cell cycle and represent a pool of potential WEE-1.3 interacting proteins that function during *C. elegans* oocyte maturation and zygotic development.

A basic tenet of reproductive biology is the conjoining of two haploid gametes, an egg and a sperm, to form a diploid zygote. One major difference between the two sexes is the method through which they create a final, functional haploid gamete. Both males and females generate haploid gametes via the meiotic cell cycle, which consists of one round of DNA replication followed by two rounds of chromosome segregation. However, meiosis is differentially regulated in the two gametes. Spermatocytes, the precursors to sperm, proceed through meiosis uninterrupted, whereas oocytes arrest during meiosis at a species-specific stage. This arrest typically occurs at prophase I, and then later when the female reaches reproductive maturity the oocytes are activated in a process referred to as meiotic maturation (Nebreda and Ferby 2000; Von Stetina and Orr-Weaver 2011). During the arrest period, the oocytes grow in size and accumulate all of the transcripts required to support meiotic maturation and fertilization (Schindler 2011).

Oocyte meiotic arrest is maintained and cell-cycle proliferation is inhibited until the oocyte receives an external hormonal stimulus that alleviates the arrest and promotes oocyte maturation. Cyclin-dependent kinases are the universal regulators of both mitotic and meiotic cell-cycle progression in eukaryotes. A complex of cyclin-dependent kinase 1 (Cdk1) and its partner cyclin B, termed maturation promoting factor (MPF), acts to drive the meiotic cell cycle (Doree and Hunt 2002; Greenstein 2005; Kishimoto 2003). The activity of MPF is regulated through phosphorylation, dephosphorylation, and cyclin degradation. During meiotic arrest in vertebrates, the MPF complex must be kept in an inactive state; this inhibition is accomplished via phosphorylations on Thr14 (T14) and Tyr15 (Y15) residues of Cdk1 by the Wee1/Myt1 family of inhibitory kinases (Schmitt and Nebreda 2002). In addition, an activating phosphorylation event occurs on Thr161 (for human Cdk1) by the Cdk-activating kinase CAK (CDK-7 in *C. elegans*) (Wallenfang and Seydoux 2002). Dephosphorylation of T14 and Y15 on CDK-1 occurs by a dual-specific phosphatase, Cdc25, resulting in a functional kinase and resumption of the meiotic cell cycle. Cyclin B is also targeted for degradation by an ubiquitin-ligase known as the anaphase promoting complex (APC), enabling the transition from meiotic metaphase I to anaphase I (Boxem 2006; Zachariae and Nasmyth 1999).

Once MPF is activated, a number of hallmark events occur that define oocyte maturation in many species. These events include nuclear envelope breakdown (NEBD), chromosome congression, rearrangement of the cortical cytoskeleton, and meiotic spindle assembly (Jones 2004; Von Stetina and Orr-Weaver 2011). In *C. elegans*, the hallmarks of oocyte maturation have been shown to occur upon sperm signaling and the presence of major sperm protein (MSP) (Miller *et al.* 2001; Singaravelu and Singson 2011; Yamamoto *et al.* 2006). Interestingly, this process of meiotic maturation is spatially restricted in the nematode gonad to the −1 oocyte, the oocyte immediately adjacent to the spermatheca. Depletion of WEE-1.3, the Myt1 inhibitory kinase ortholog, in *C. elegans* results in precocious oocyte maturation *in vivo* (Burrows *et al.* 2006). The precociously maturing oocytes exhibit premature NEBD, chromosome over-congression where the chromosomes have coalesced into a single mass of chromatin, aberrant meiotic spindle organization, and premature oocyte-to-embryo transition as evidenced by the premature relocalization of MBK-2 in the WEE-1.3–depleted oocytes (Burrows *et al.* 2006). These oocytes are ovulated and do encounter sperm; however, they are fertilization-incompetent and the animals are infertile (Burrows *et al.* 2006). Similarly, *in vitro* antibody depletion of Myt1 in *Xenopus* oocytes results in precocious NEBD (Nakajo *et al.* 2000).

In this study, we sought to expand on the role that WEE-1.3 plays in *C. elegans* oocyte maturation and further investigate the precociously maturing oocytes exhibited upon depletion of WEE-1.3. We found that WEE-1.3 depletion results in a premature oocyte-to-embryo transition as demonstrated by the relocalization of maternal proteins within the oocyte to embryonic patterns of distribution. In addition, the WEE-1.3–depleted oocytes have undergone embryonic gene activation (EGA), despite the fact that oocytes are normally transcriptionally quiescent and wild-type embryonic transcription is not reported to begin until the four-cell embryo (Biedermann *et al.* 2009; Seydoux *et al.* 1996). Finally, we performed an RNAi suppressor screen to identify factors that, when co-depleted with WEE-1.3, resulted in restoration of fertility. The 44 identified factors are potentially novel regulators and interactors with WEE-1.3, but also could be regulators and interactors with CDK-1.

## Materials and Methods

### Strains

Wild-type *C. elegans* was Bristol strain N2. All strains were grown under standard conditions at 20° (Brenner 1974), except the WEE-1.3–tagged transgenes, which were grown at 24° to visualize expression. A list of all the strains used can be found in Table 1. Some nematode strains used in this work were provided by the *Caenorhabditis* Genetics Center, which is funded by the NIH National Center for Research Resources (NCRR). The FIB-1::GFP transgenic strain (COP262) was generated using a custom transgenic service (Knudra Transgenics, Salt Lake City, UT).

**Table 1:** Nematode strains used in this study

Name	Description	Genotype	Reference
AG221	WEE-1.3::GFP	*unc-119(ed3)*; *avIs147[pAA34 (unc-119(+) + wee-1.3* prom::WEE-1.3::GFP::*wee-1.3* 3′UTR*]*	This study
AG222	GFP::WEE-1.3	*unc-119(ed3)*; *avEx148[pAA10(unc-119(+) + wee-1.3* prom::GFP::WEE-1.3::*wee-1.3* 3′UTR*)]*	This study
VC465	*wee-1.3* deletion	*wee-1.3(ok729)/mIn1[mIs14 dpy-10(e128)]* (1168bp deletion)	CGC
JH1576	GFP::MBK-2	*unc-119(ed3)*; *axIs1140 [pJP1.02(unc-119(+) + pie-1* prom::GFP::MBK-2)*]*	Pellettieri *et al.* 2003
AD200	GFP::EGG-3	*unc-119(ed3)*; *asIs1[unc-119(+) + pie-1* prom::GFP::EGG-3*]*	Maruyama *et al.* 2007
RT688	CAV-1::GFP	*unc-119(ed3)*; *pwIs28[pie-1* prom::CAV-1::GFP:: *pie-1* 3′UTR *+ unc-119(+)]*	Sato *et al.* 2006
AD265	GFP::CHS-1	*unc-119(ed3)*; *nnIs2[unc-119(+) + pie-1* prom::GFP::CHS-1*]*	Maruyama *et al.* 2007
JJ2101	PGL-1::GFP	*unc-119(ed3)*; *zuIs242[unc-119(+) + nmy-2* prom::PGL-1::GFP::*nmy-2* 3′UTR*]*	Wolke *et al.* 2007
TX189	OMA-1::GFP	*unc-119(ed3)*; *teIs1[pRL475(oma-1* prom::OMA-1::GFP*) + pDPMM016(unc-119+)]*	Lin 2003
AG212	CBD-1::mCherry	*unc-119(ed3)*; *avIs143 [pDNL10 (unc-119(+) + cbd-1* prom::CBD-1::mCherry::*cbd-1* 3′UTR*)]*	This study
OCF22	mCherry::NPP-1	*unc-119(ed3)*; *ocfIs5[unc-119(+) + pie-1* prom::mCherry::NPP-1::*pie-1* 3′UTR*]*	Joseph-Strauss *et al.* 2012
OCF15	mCherry::SP12	*unc-119(ed3)*; *ocfIs2[unc-119(+) + pie-1* prom::mCherry::SP12::*pie-1* 3′UTR*]*	Joseph-Strauss *et al.* 2012
AG223	WEE-1.3::GFP ; mCherry::NPP-1	*unc-119(ed3)*; *avIs147[pAA34 (unc-119(+) + wee-1.3* prom::WEE-1.3::GFP::*wee-1.3* 3′UTR*]*; *ocfIs5[unc-119(+) + pie-1* prom::mCherry::NPP-1::*pie-1* 3′UTR*]*	This study
AG224	WEE-1.3::GFP ; mCherry::SP12	*unc-119(ed3)*; *avIs147[pAA34 (unc-119(+) + wee-1.3* prom::WEE-1.3::GFP::*wee-1.3* 3′UTR*]*; *ocfIs2[unc-119(+) + pie-1* prom::mCherry::SP12:: *pie-1* 3′UTR*]*	This study
COP262	FIB-1::GFP	*unc-119(ed3)*; *knuSi221*[*pNU162* (*fib-1* prom::FIB-1::eGFP::*fib-1* 3′ UTR + *unc-119(+)*]	This study
OCF1	mCherry::Histone H2B	*unc-119(ed3)*; *ltIs37 [pAA64 (unc-119(+) + pie-1* prom::mCherry::*his-58* 3′UTR)]	Golden *et al.* 2009
AG229	FIB-1::GFP ; mCherry:: Histone H2B	*unc-119(ed3)*; *knuSi221*[*pNU162* (*fib-1* prom::FIB-1::eGFP::*fib-1* 3′ UTR + *unc-119(+)*]; *ltIs37 [pAA64 (unc-119(+) + pie-1* prom::mCherry::*his-58* 3′UTR)]	This study

### Plasmid construction

All plasmids were constructed using PCR from genomic N2 DNA and the Gateway cloning technology (Invitrogen, Grand Island, NY). The sequences of all entry clones were confirmed. The final plasmids generated were pAA10 (*wee-1.3* prom::GFP::WEE-1.3::*wee-1.3* 3′UTR) and pAA34 (*wee-1.3* prom::WEE-1.3::GFP::*wee-1.3* 3′UTR). The promoter sequence utilized in each construct is as follows: 1047 bp (for pAA10) and 957 bp (for pAA34) upstream of the *wee-1.3* translational start site. The *wee-1.3* 3′UTR is annotated in WormBase (release WS232) as being 446 nucleotides long. We utilized a slightly longer piece of genomic DNA in the translational reporters to ensure proper expression *in vivo* (523 bp downstream of the stop codon). The PCR products were cloned into the entry vectors (Invitrogen, Grand Island, NY) pDONR(P4-P1r) and pDONR(P2r-P3) as described in Supporting Information, Table S1 via a Gateway BP reaction. pAA10 was generated through a MultiSite Gateway LR reaction utilizing the following plasmids: pAA11, pCR110, pAA15, and pCR319. pAA34 was generated through a MultiSite Gateway LR reaction utilizing the following plasmids: pAA32, pCR110, pAA13, and pCR319. A description of all plasmids and primer sequences used can be found in Table S1.

### CBD-1::mCherry construction

The entry clone containing the *cbd-1* promoter and *cbd-1* coding sequences (exons and introns) was made as follows. N2 genomic lysates were PCR-amplified with primers B4F2 and B1R3 (see Table S1 for primer sequences). The 5.6-kb fragment was recombined into pDONR P4P1R. This construct has 1169 bp of 5′ UTR sequence. Sequencing revealed an error at nucleotide #1921 (T→C nucleotide change; V→A amino acid change), which was corrected with the QuikChange Mutagenesis kit (Agilent Technologies, Santa Clara, CA). The entry clone containing the *cbd-1* 3′ UTR was made as follows. N2 genomic lysates were PCR-amplified with primers B2rF2 and B3R1. The 389-bp PCR fragment was recombined into pDONR P2RP3. The expression clone was constructed by performing a Gateway LR reaction with the above two entry clones, pCR347 and pCR319.

### FIB-1::GFP construction

DNA coding for the *fib-1* promoter, genomic sequence, and 3′ UTR was inserted into pCFJ151 (ttTi5606) targeting vector to make pNU162 (*fib-1* prom::FIB-1::eGFP::*fib-1* 3′UTR). pNU162 was microinjected into EG6699 (ttTi5605) strain and MosSCI transgenic capture was performed with mCherry markers. Isolates were screened, a single copy insertion at Mos locus was determined via PCR, and the line COP262 was generated (knuSi221 [pNU162 (fib-1p::FIB-1::eGFP::fib-1 3′UTR, *unc-119(+)*)] II ; *unc-119 (ed3)* III). All services were performed by Knudra Transgenics (Salt Lake City, UT).

### *C. elegans* transformation

All transgenic lines, except for the FIB-1::GFP line described above, were generated by microparticle bombardment as previously described, except that animals were grown in liquid culture before transformation (Praitis *et al.* 2001; Stein *et al.* 2010). For each construct, expression was analyzed and found to be similar in at least five independent lines. Specifically, a strain containing an integrated transgene, *avIs147*, and a strain containing an extrachromosomal array, *avEx148*, are detailed in this article. To determine if the transgenes could rescue the absence of WEE-1.3, *avIs147* (WEE-1.3::GFP) was crossed to the *wee-1.3* deletion line (VC465) to obtain a line that is homozygous for the deletion and contains the transgene.

### RNA interference

RNAi was performed via feeding as described using HT115(DE3) bacteria seeded on MYOB plates containing 2 mM IPTG and 25 μg/mL carbenicillin (Timmons and Fire 1998). RNAi constructs were obtained from the Open Biosystems library (Huntsville, AL), and the identity of each suppressor clone was verified by sequencing. L4 hermaphrodites were fed for 20–24 hr at 24° and then moved to a new RNAi plate for another 20–24 hr (3 animals per plate). At the end of the second 24-hr period, hermaphrodites were removed and either discarded or imaged depending on the experiment. The second-day plate was scored 1 d later to determine brood size and hatching data. As a control, worms were fed bacteria expressing double-stranded RNA (dsRNA) against *smd-1(F47G4.7)*, which does not produce a visible phenotype. For combinatorial RNAi, bacterial cultures expressing the dsRNA were grown separately to saturation and mixed in equal volume amounts immediately prior to seeding the plates. Brood size analysis was conducted by totaling the number of embryos and larvae on the indicated plates.

### Live imaging

Adult hermaphrodites of the appropriate genotype were picked onto a slide with a 1% agar pad containing a drop of M9/0.2 mM levamisole. A coverslip was then placed on top of the drop and sealed with nail polish. Paralysis was allowed to set in for 10 min before imaging proceeded. Animals were then imaged using the techniques described below in the *Microscopy* section.

### Immunofluorescence

Adult hermaphrodites were picked into 100 μL of PBS/0.1% Tween-20 (PBTw) in a deep watch glass, levamisole was added to a final concentration of 0.2 mM, and then the animals were dissected by cutting off the heads at the level of the pharynx using 25-gauge syringe needles, such that at least one gonad arm extruded completely. The animals were fixed in 2 mL of 3% PFA solution (3% formaldehyde/0.1 M K_2_HPO_4_/0.1 M KH_2_PO_4_) for 10 min. After fixation, an equal volume of PBTw was added and the gonads were transferred to a 5-mL glass conical tube before being centrifuged for 1 min on low speed in a clinical benchtop centrifuge. Supernatant was discarded, and then the animals were washed twice with PBTw and post-fixed in ice-cold 100% methanol for a minimum of 5 min. The tube was filled with PBTw, centrifuged as described above, supernatant was removed, and animals were washed three times with PBTw. After the third wash, animals were transferred to a small glass culture tube (Kimble Chase #73500650, Vineland, NJ) and allowed to settle before most of the liquid was aspirated and samples were blocked in PBTw containing 30% normal goat serum (NGS) for 1 hr at room temperature. The block was removed, samples were washed in PBTw, primary antibodies were added, and samples were put at 4° for 16–20 hr. The supernatant was removed, samples were washed three times in PBTw for 5 min each, and secondary antibodies were added for 2 hr at room temperature in the dark. Removal of the supernatant was followed by three 5-min washes in PBTw, with the last wash containing 100 ng/mL DAPI. Vectashield (Vector Laboratories, Burlingame, CA) and the samples were transferred to a large 2% agar pad on a standard microscope slide. Excess liquid was withdrawn with a capillary, and an eyelash hair was used to manipulate and position gonads. A large coverslip was placed on top of the samples and sealed with nail polish. Slides were allowed to rest for at least 1 hr in the dark before they were imaged. Primary antibodies were diluted in PBTw as follows: rabbit anti-phospho-histone H3(Ser10) (1:200; Upstate Biotechnology, Waltham, MA), mouse anti-Nop1p (1:100; EnCor Biotech, Alachua, FL), and mouse anti-WEE-1.3 (1:500; this study). Secondary antibodies were Alexa Fluor 448–conjugated or Alexa Fluor 568–conjugated goat anti-rabbit or goat anti-mouse (1:1000; Invitrogen, Grand Island, NY).

### Generation of anti-WEE-1.3 antibody

A monoclonal mouse antibody to WEE-1.3 (ab4D5) was generated using a custom antibody service (Abmart, Shanghai, CH) to the C-terminal peptide sequence DLPRMPVLNF.

### Microscopy

Fluorescent images of live or fixed samples were captured either using spinning-disk confocal microscopy as described previously in Golden *et al.* (2009) or using a Nikon Ti-E-PFS inverted microscope equipped with a 60× 1.4NA Plan Apo Lamda objective. The Ti-E-PFS system is outfitted with a Yokogawa CSU-X1 spinning disk unit, a self-contained four-line laser module (excitation at 405, 488, 561, and 640 nm), and Andor iXon 897 EMCCD camera. Confocal images were acquired using Openlab 4.0 or NIS-Elements and processed using ImageJ 1.38X and Adobe Photoshop CS5 software. All images shown are single focal planes unless noted.

### RNAi suppressor screen

1874 RNAi clones within the Open Biosystems collection that had been reported on WormBase (release WS232) as having an embryonic lethal phenotype (EMB) were screened to determine if they suppressed the WEE-1.3-depletion phenotype (Harris *et al.* 2010; Kamath *et al.* 2003; Sonnichsen *et al.* 2005). RNAi was performed as described above. For combinatorial RNAi, bacterial cultures expressing the dsRNA were grown separately and mixed in equal volume amounts immediately before seeding the plates. Controls included were: control (*smd-1*) RNAi; *cdk-1* RNAi; *wee-1.3* RNAi; co-depletion of *wee-1.3* and *smd-1*; and co-depletion of *wee-1.3* and *cdk-1*.

In phase I, suppression was scored visually based on appearance of progeny on the plate. Clones that failed to suppress had plates with no progeny; weak suppressors had plates with <10 embryos; moderate suppressors had plates with <50 embryos and, in some cases, a few larvae; and strong suppressors had plates with >50 larvae that, in some instances, developed into adult animals. All candidate suppressors were then retested to ensure accuracy in visual scoring, tested to see if they were global suppressors of RNAi utilizing combinatorial RNAi with *lit-1* and each potential suppressor, and sequenced using an M13 forward primer to verify their identities along with a subset of nonsuppressing candidates (n = 42). Approximately 6% of the 193 sequenced clones from the library did not show any homology to *C. elegans* genes by BLAST. All unconfirmed clones are noted in Table S2.

In phase 2, quantification of 52 of the 57 identified moderate and strong candidate suppressors was conducted (see Table S2, “Quantified Suppressors”). RNAi clones were re-transformed into HT115(DE3) and RNAi was conducted as above, with the exception that single L4 hermaphrodites (n = 12) were placed on individual plates to determine brood size per individual animal. At least three independent experiments were conducted for each condition, and then the average brood size, percent hatching, and SEM for each RNAi treatment was determined as described above. The total number of hermaphrodites tested for each RNAi treatment was between 24 and 100 animals. Statistics were performed using a Student’s *t*-test and comparing candidate suppressor brood results with the brood exhibited by *wee-1.3/control* RNAi-treated animals.

### Gene ontology analyses

Functional enrichment was assessed using the DAVID database (http://david.abcc.ncifcrf.gov/). The suppressors were analyzed for GOTERM_BP_5 and GOTERM_CC_5 through functional annotation clustering using medium classification stringency with a similarity threshold of 0.50. The statistical significance threshold level for all gene ontology enrichment analyses was *P* < 0.05 (Huang *et al.* 2009a,b).

### Quantification of mRNAs in dissected gonads

Total RNA was isolated from ∼100 dissected gonads per RNAi treatment using the Arcturus PicoPure RNA Isolation Kit (Applied Biosystems, Foster City, CA). Dissected gonads consisted of the gonad region from the distal tip to the spermatheca, but did not contain any portion of the uterus or embryos. Isolation included the optional DNase treatment to remove DNA contamination. Real-time PCR reactions were performed in triplicate using 5 ng of template RNA, Quantifast SYBR Green RT-PCR Kit (Qiagen, Valencia, CA), and appropriate primers in an ABI 7900HT Fast Real-Time PCR detection system (Applied Biosystems, Carlsbad, California) according to the manufacturer’s instructions. Amplicons were designed, when possible, to span one or more introns to avoid amplification of the target gene in genomic DNA. For primer sequences, see Table S1. PCR reactions were performed as follows: 10 min at 50°, 5 min at 95°, then 40 cycles of 10 sec at 95° and 30 sec at 60°. Each PCR reaction then concluded with determination of the melting curve of the amplicon to verify amplification of only one product. A no reverse transcriptase control was performed for each tested RNA and a nontemplate control was performed for each primer. Average Ct values were determined by the SDS v2.3 software for each primer pair. Fold change was calculated using the comparative Ct (ΔΔCt) method where values for a particular transcript were normalized to an endogenous control within each sample (*act-1* mRNA levels) and then normalized to the calibrator sample (*control RNAi* gonads). Error bars show the SEM for the replicates.

## Results

### WEE-1.3 is ubiquitously expressed and perinuclear

**Figure 1:**
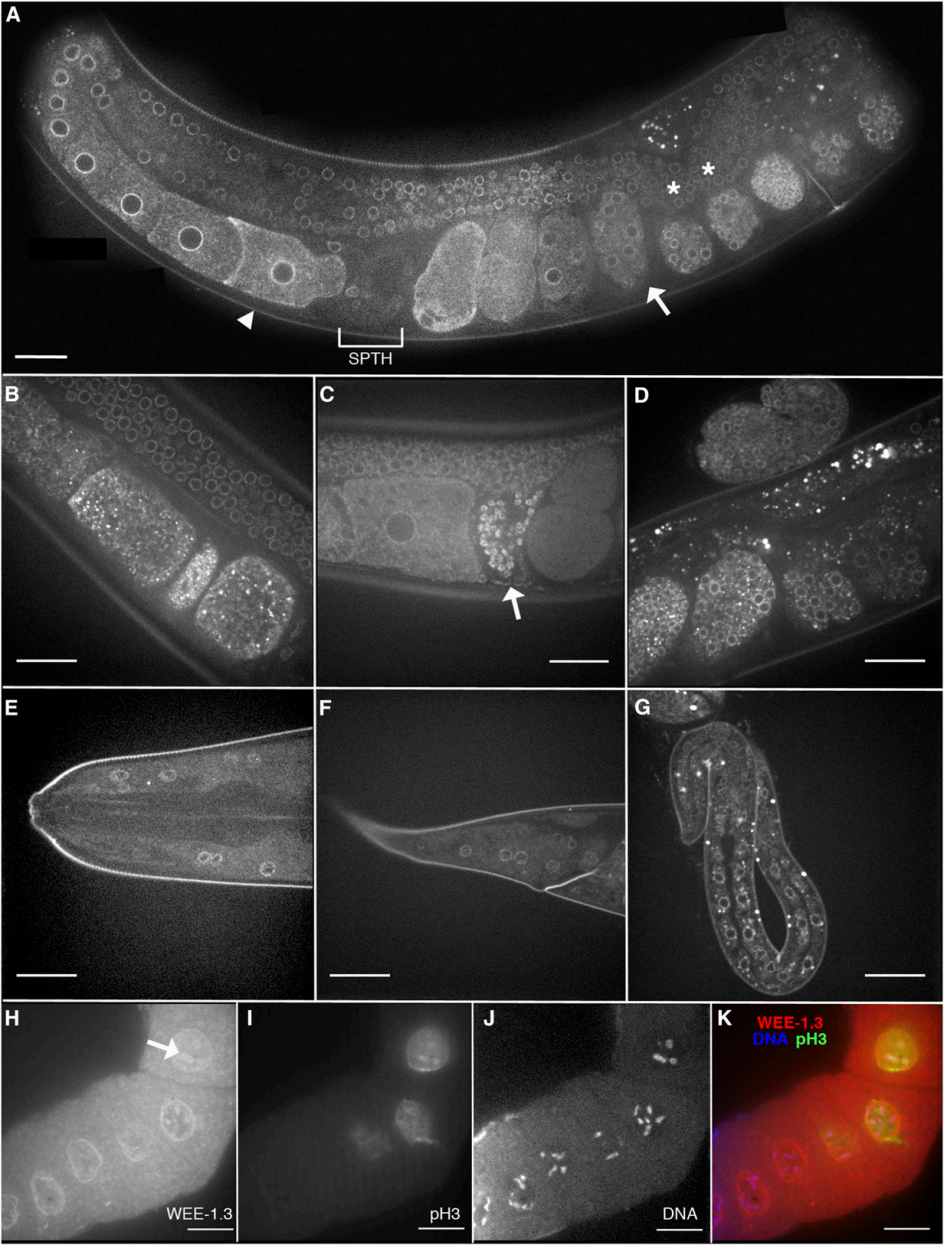
WEE-1.3 is expressed throughout the adult soma, germline, and embryos. (A–G) Fluorescence micrographs of live animals expressing integrated transgene *avIs147 [unc-119(+) + pAA34(wee-1.3* prom::WEE-1.3::GFP::*wee-1.3* 3′UTR*)]* or an extra chromosomal array of *avEx148 [unc-119(+) + pAA10(wee-1.3* prom::GFP::WEE-1.3 *+ wee-1.3* 3′UTR*)]*. (A) Adult hermaphrodite exhibits perinuclear WEE-1.3::GFP expression in the germline from the distal tip (asterisk) to the proximal oocytes (arrowhead) and in developing embryos (arrows) [*avIs147 (pAA34)*]. Spermatheca (SPTH) is indicated by the bracketed region. Note the distal tips from both gonad arms are shown in this image. (B) Punctate expression of WEE-1.3::GFP on the surface of oocytes in the proximal germline of a rescued *wee-1.3* deletion line [*wee-1.3(ok729)*; *avIs147 (pAA34)*]. (C) Sperm expression (arrow) of GFP::WEE-1.3 in the spermatheca of an *avEx148* animal (pAA10). (D) Perinuclear and punctate expression of WEE-1.3::GFP throughout developing embryos of a rescued *wee-1.3* deletion line [*wee-1.3(ok729)*; *avIs147 (pAA34)*]. (E) Somatic head nuclei expressing WEE-1.3::GFP in a rescued *wee-1.3* deletion line [*wee-1.3(ok729)*; *avIs147 (pAA34)*]. (F) Somatic tail nuclei expressing WEE-1.3::GFP in a rescued *wee-1.3* deletion line [*wee-1.3(ok729)*; *avIs147 (pAA34)*]. (G) Larval somatic expression of WEE-1.3::GFP in a rescued *wee-1.3* deletion line [*wee-1.3(ok729)*; *avIs147 (pAA34)*]. (H–K) Z-stack projections of confocal images of gonads dissected from wild-type mothers, fixed, and co-stained with antibodies against WEE-1.3 (H, red in K), phospho-histone H3 (Ser10) (I, green in K), and DNA (J, blue in K). The arrow in (H) indicates WEE-1.3 expression that coats the diakinetic chromosomes in mature oocytes. Scale bars are approximately 20 μm.

We examined the expression pattern of WEE-1.3 by constructing translational fusion transgenes and generating transgenic animals. Both N- and C-terminally tagged GFP and mCherry transgenes that contained the endogenous *wee-1.3* promoter, full-length *wee-1.3* sequence, and *wee-1.3* 3′UTR were created, and multiple transgenic lines were generated by microparticle bombardment (Praitis *et al.* 2001). In total, 12 extrachromosomal lines (7 N-terminally tagged, 5 C-terminally tagged) and 10 integrated lines (6 N-terminal tagged, 4 C-terminally tagged) were generated, of which 11 and 7, respectively, resulted in strong expression (data not shown). All expressing transgenes exhibited similar patterns; as such, only two lines are detailed here, *avIs147* and *avEx148*, henceforth referred to as WEE-1.3::GFP or GFP::WEE-1.3, respectively. WEE-1.3::GFP expression is perinuclear throughout the adult soma, germline, and within developing embryos (Figure 1). Perinuclear expression is observed from the distal tip of the germline to the mature proximal oocytes (Figure 1A, asterisk and arrowhead, respectively). It then continues to be expressed in developing embryos within the uterus of the animal (Figure 1A arrow, Figure 1D). In addition to the perinuclear expression within the germline, there is some diffuse cytoplasmic expression within developing oocytes and embryos, expression on the membrane within developing oocytes and embryos, and distinct punctae on the surface of the oocytes (Figure 1B) and within embryos (Figure 1D). The spermatheca of hermaphrodites of *avEx148*, along with other extrachromosomal lines (7 of 12) and some integrated lines (3 of 7), exhibit GFP::WEE-1.3 within sperm (Figure 1C). In the somatic tissues of the adult, perinuclear WEE-1.3::GFP expression is observed throughout the adult, including nuclei of the head (Figure 1E) and tail (Figure 1F). The nuclei of many cell types in the developing larvae, including the cells of the developing intestine (Figure 1G), also exhibit WEE-1.3::GFP expression. Importantly, *avIs147* rescued the *wee-1.3(ok729)* deletion mutant to viability and therefore is believed to make functional WEE-1.3 protein (Figure 1, B, D–G).

Subcellular localization of WEE-1.3::GFP was shown to be perinuclear through colocalization studies with a tagged version of a known nucleoporin protein, mCherry::NPP-1, that is localized to the nuclear envelope (Figure S1, A–C) (Joseph-Strauss *et al.* 2012; Schetter *et al.* 2006). In addition, WEE-1.3::GFP appears to be localized to the endoplasmic reticulum (ER) through colocalization with a tagged version of a known ER marker, SP12 (Figure S1D–I) (Rolls *et al.* 2002). WEE-1.3::GFP does not colocalize with the entire endoplasmic reticulum, as was shown through RNAi experiments involving *lpin-1*. In animals fed control RNAi, the ER appears as a network of fine tubules with occasional small patches (Figure S1E), and WEE-1.3::GFP has a substantial amount of overlap with the ER marker SP12 (Figure S1F). However, when the ER is disrupted using RNAi to *lpin-1*, the formation of ER-like membrane sheets, patches, and vesicle-like structures appear (Figure S1H) (Golden *et al.* 2009). In *lpin-1*–RNAi treated animals, WEE-1.3::GFP does not colocalize with all regions of the expanded and disorganized ER membrane (Figure S1I).

### WEE-1.3 coats the diakinetic chromosomes

We generated a mouse monoclonal antibody to WEE-1.3 (mAb4D5) that confirmed all the expression patterns indicated by the various integrated and extrachromosomal WEE-1.3 transgenic lines (data not shown). In addition, this antibody appears to coat the diakinetic chromosomes found in the mature oocytes (Figure 1H, arrow). This can be seen through co-staining for phospho-histone H3 (Ser10), a known marker for condensed chromatin that is expressed in only mature oocytes, and DAPI for DNA (Figure 1K). The WEE-1.3 expression is observed within the nucleus of mature oocytes and appears to surround the individual chromosomes.

### Precocious oocyte-to-embryo transition occurs in animals depleted of WEE-1.3

After an oocyte has been fertilized, a dynamic process referred to as the oocyte-to-embryo (OTE) transition occurs. During the OTE transition, a number of proteins (such as EGG-4/5, EGG-3, MBK-2, and CHS-1) exhibit dramatic changes in their localization pattern (Parry and Singson 2011). EGG-4/5 proteins are cortical in late oocytes and become diffusely cytoplasmic in embryos. EGG-3, MBK-2, and CHS-1 are also cortical in late oocytes; however, these proteins relocalize to discrete cytoplasmic punctae in the embryo.

Depletion of WEE-1.3 has been previously shown to cause premature relocalization of EGG-4/5, EGG-3, CHS-1, and MBK-2 in the oocytes from the cortex to a diffuse cytoplasmic pattern (EGG-4/5) or to discrete cytoplasmic punctae (EGG-3, CHS-1, and MBK-2) (Cheng *et al.* 2009; Stitzel *et al.* 2007). Conversely, the CAV-1 protein, which is localized to punctae in oocytes and relocalizes to the cortex in embryos, was reported to not exhibit premature relocalization to the cortex of oocytes on WEE-1.3 depletion (Bembenek *et al.* 2007). To further characterize the precocious oocyte maturation phenotype in animals depleted of WEE-1.3, we examined additional proteins that had been identified as having distinct localization patterns in the oocyte *vs.* the embryo.

**Figure 2:**
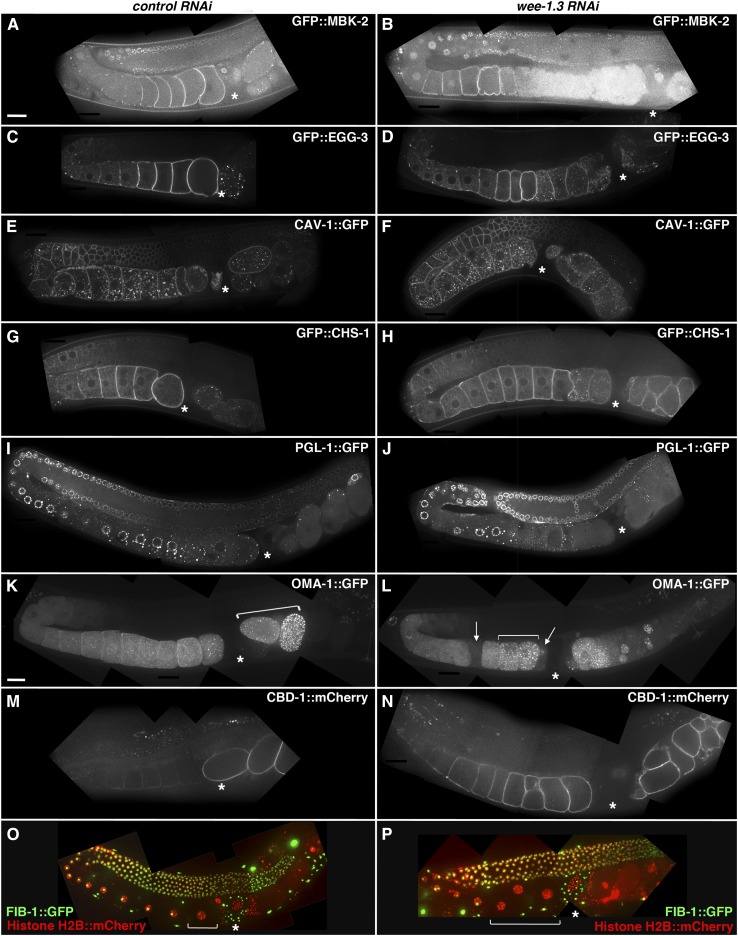
Precocious oocyte-to-embryo transition upon WEE-1.3 RNAi depletion. (A–N) Single confocal images of live wild-type (A, C, E, G, I, K, and M) or *wee-1.3(RNAi)* (B, D, F, H, J, L, and N) hermaphrodites expressing GFP::MBK-2 (A, B), GFP::EGG-3 (C, D), CAV-1::GFP (E, F), GFP::CHS-1 (G, H), PGL-1::GFP (I, J), OMA-1::GFP (K, L), or CBD-1::mCherry (M, N). Brackets in (K and L) denote OMA::GFP localizing to discrete punctae. Arrows in (L) denote oocytes undergoing premature degradation of OMA-1::GFP. (O and P) Z-stack projections of live images of FIB-1::GFP; Histone H2B::mCherry hermaphrodites (FIB-1 in green and histones in red). Asterisk denotes the position of the spermatheca with oocytes to the left of the spermatheca and embryos to the right. Bracket in (O and P) denote mature oocytes. Scale bar in (A) applies to (A–J) and (M–P), and is approximately 20 μm. Scale bar in (K) applies to (K and L) and is approximately 20 μm. More than 20 animals were observed and imaged for each condition, and a representative image is shown.

We were able to confirm that GFP::MBK-2, GFP::EGG-3, and GFP::CHS-1 all relocalized precociously to internal punctae in *wee-1.3(RNAi)* oocytes, whereas CAV-1::GFP appeared normal in treated oocytes (Figure 2, A–H). PGL-1, a known P-granule component, is localized to cytoplasmic punctae that concentrate perinuclearly in oocytes and very early embryos. This protein then undergoes degradation in all cells of the developing embryo except the P4 (germline) blastomere (Kawasaki *et al.* 2004). PGL-1::GFP was degraded prematurely in the oocytes of *wee-1.3(RNAi)* animals compared with oocytes of control animals (Figure 2, I–J). OMA-1 is another protein that is expressed throughout the cytoplasm of maturing oocytes (with levels peaking in oocytes undergoing maturation). Then, in early embryos, OMA-1 becomes localized to discrete punctae before it is rapidly degraded by the four-cell stage (Detwiler *et al.* 2001). Upon depletion of WEE-1.3, we observed the premature appearance of a large number of discrete punctae in the oocytes (Figure 2, K and L, compare brackets) and occasional oocytes undergoing premature degradation of OMA-1::GFP (Figure 2L, arrows).

The chitin-binding domain protein CBD-1, a component of the eggshell, has been previously reported to localize around oocytes in the proximal germline (Johnston *et al.* 2010). Our data using a CBD-1::mCherry transgenic animal generated for this study confirm that localization but also demonstrate that the level of CBD-1 on the cortex of the oocytes is substantially reduced compared with the level observed after fertilization and establishment of the eggshell in the developing embryos (Figure 2M). Interestingly, CBD-1::mCherry is prematurely expressed in WEE-1.3–depleted oocytes when compared with expression in control oocytes (Figure 2, M and N). Taken together, the above results suggest that the oocytes formed upon WEE-1.3 depletion exhibit multiple hallmarks of having precociously proceeded through the oocyte-to-embryo transition.

To identify the earliest time point at which this precocious oocyte-to-embryo transition occurs, we generated an animal that possessed both fluorescently tagged nucleoli and histones (FIB-1::GFP; Histone H2B::mCherry) (Figure 2, O and P). The absence of a nucleoli, as evident by no Nop1p staining, and the presence of condensed chromosomes, as evident by positive staining for phospho-histone H3 (pH 3), are two markers by which precocious oocyte maturation has been characterized and observed in WEE-1.3–depleted animals (Burrows *et al.* 2006). The FIB-1::GFP; Histone H2B::mCherry animals allowed us to determine via live imaging that after 16 hr of feeding of WEE-1.3 RNAi, the nucleoli within a number of proximal oocytes begins to break-down, as observed by an absence of FIB-1::GFP spots within the nucleus (Figure 2P, bracket). This is in stark contrast to what is observed in control animals, where only the −1 oocyte lacks a nucleolus (Figure 2O, bracket). This animal affords us the opportunity to observe when precocious oocyte maturation is starting *vs.* examining the final output of precocious oocyte maturation, infertility.

### Embryonic gene activation occurs in oocytes of animals depleted of WEE-1.3

The premature relocalization of a subset of proteins within the WEE-1.3-depleted oocytes led us to question whether those oocytes had undergone embryonic gene activation (EGA) (Baroux *et al.* 2008; Edgar *et al.* 1994). Oocytes are transcriptionally quiescent, and it is only after fertilization, upon the oocyte-to-embryo transition, that transcription begins. This process is termed embryonic gene activation. In *C. elegans*, embryos do not begin transcribing mRNAs until they have reached the four-cell embryo stage, and EGA is characterized by the transcription of several very early transcripts (*e.g.*, *vet-1*, *vet-4*, and *vet-6*) (Schauer and Wood 1990; Seydoux *et al.* 1996). To determine if WEE-1.3–depleted oocytes exhibit precocious onset of EGA, we performed quantitative RT-PCR to compare the levels of *vet-1*, *vet-4*, and *vet-6* mRNAs in gonads dissected from *control(RNAi)* or *wee-1.3(RNAi)* animals.

**Figure 3:**
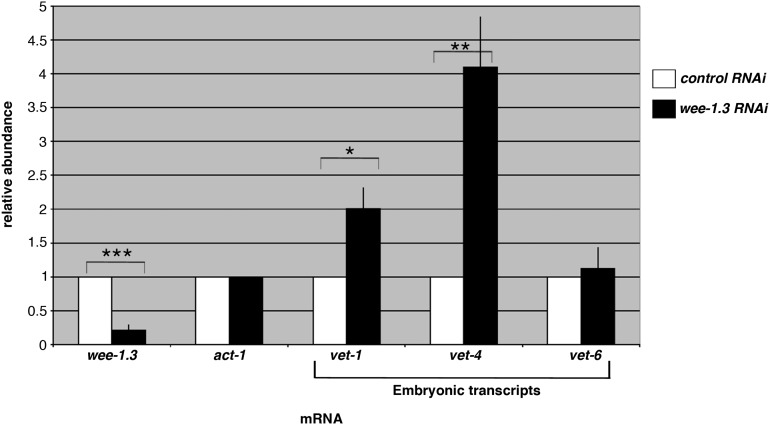
Depletion of WEE-1.3 results in precocious onset of embryonic gene activation. The expression of the three mRNAs, *vet-1*, *vet-4*, and *vet-6*, is normally restricted to the early embryo; however, in gonads subjected to *wee-1.3(RNAi)* they are expressed precociously in the gonad. The graph shows the relative amounts of the indicated mRNAs, determined by quantitative RT-PCR, found in the gonads of adults subjected to either control or *wee-1.3* RNAi. Relative abundance for each target gene on *wee-1.3* RNAi treatment was calculated using the comparative Ct (ΔΔCt) method in which values were first normalized to *act-1* mRNA levels and then normalized to the calibrator sample (control RNAi gonads). Each bar represents the mean of three independent biological replicates, and the error bars represent SEM. Statistics were performed using a Student’s *t*-test. *P-value <0.05; **P-value <0.01; and ***P-value ≤0.0001.

To determine that we were successfully depleting animals of WEE-1.3, we first analyzed the abundance levels of *wee-1.3* mRNA in the gonads of *control(RNAi)* and *wee-1.3(RNAi)* animals and found a 4.7-fold decrease in mRNA levels upon treatment with *wee-1.3(RNAi)* (Figure 3). We found that *wee-1.3(RNAi)* gonads had higher levels of *vet-1* and *vet-4* than the *control(RNAi)* gonads, with two-fold and four-fold enrichment, respectively (Figure 3). This suggests that the unfertilized oocytes have undergone precocious EGA. Notably, the levels of *vet-6* mRNA were not significantly different between the *control(RNAi)* and *wee-1.3(RNAi)* gonads (Figure 3), suggesting that *wee-1.3(RNAi)*-treated gonads express some, but not all, of the mRNAs normally transcribed in the early embryo.

### An RNAi screen identifies 44 suppressors of the WEE-1.3 depletion phenotype

Previously, we described the WEE-1.3 RNAi depletion phenotype as infertility. RNAi-treated hermaphrodites exhibit precocious oocyte maturation as characterized by a variety of oocyte maturation markers (our results; Burrows *et al.* 2006). This phenotype could be suppressed by the co-depletion of CDK-1 and WEE-1.3, resulting in broods of more than 100 one-cell arrested embryos, whereas co-depletion of an unrelated dsRNA (*zyg-1*) did not suppress the infertility of WEE-1.3 depletion (Burrows *et al.* 2006). To identify other WEE-1.3 interactors and additional genes involved in oocyte maturation, we performed an RNAi suppressor screen using the embryonic lethal (EMB) clones in the OpenBiosystem RNAi library (Reboul *et al.* 2003; Rual *et al.* 2004). EMB clones were chosen as a subset of the genome to screen because null mutants of *wee-1.3* are late embryonic or L1 lethal, and we reasoned that any interacting gene might also be involved in early embryogenesis and could potentially be embryonic lethal when depleted. Eventually, after screening this subset of the genome, we intend to go back and screen the entirety of the genome in a similar manner. We specifically chose to use a double RNAi screen to identify interactors and/or regulators of WEE-1.3 due to the fact that there are no hypomorphic alleles of *wee-1.3* to suppress and dominant mutants for *wee-1.3* have only a sperm phenotype (Lamitina and L'Hernault 2002). In addition, RNAi of *wee-1.3* in L4 animals is the only method through which the precocious oocyte maturation and sterile phenotype can be observed because WEE-1.3 is required for larval development.

**Figure 4:**
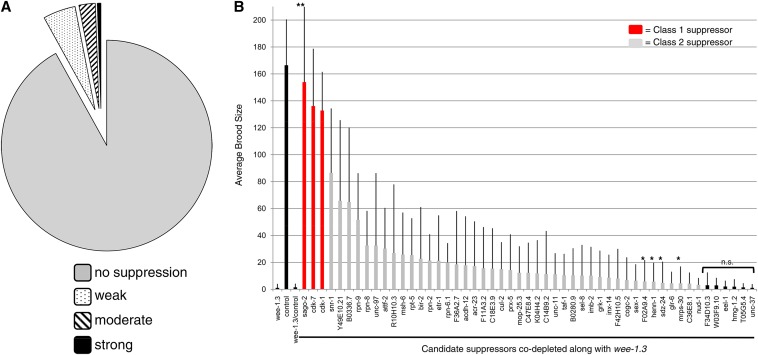
Suppression screen of the WEE-1.3 RNAi infertility results in the identification of 44 candidate suppressors. (A) Pie chart summarizing the suppression screen data in which 1874 RNAi clones were screened to identify suppressors of the WEE-1.3 infertility phenotype. 92.0% (n = 1723) failed to suppress, 5.0% (n = 94) weakly suppressed, 2.5% (n = 47) moderately suppressed, and 0.5% (n = 10) strongly suppressed the WEE-1.3 RNAi depletion infertility phenotype. (B) Quantification of the degree of suppression of 51 of the moderate and strong candidates identified in Figure 4A. Average brood size per individual mother treated with *wee-1.3*(RNAi), *control*(RNAi), or co-depleted of *wee-1.3* and *candidate suppressor* RNAi. Class 1 suppressors are denoted by a red bar and class 2 suppressors are denoted by a gray bar. Each bar represents the average of at least three independent experiments, and the errors bars represent SEM. The total n per each condition was at least 24 and at most 100 animals. Statistics were performed using a Student’s *t*-test; all values are *P*-value <0.005 when compared with the brood exhibited by *wee-1.3/control* RNAi-treated animals, except *P-value <0.05; n.s. indicates not significant. **Indicates a global RNAi suppressor.

We screened 1874 EMB clones from the library, which represent 1722 unique genes, or ∼8%, of the 20,553 protein-coding genes of *C. elegans* (as of WormBase WS232). The genes we screened were ones that had been classified as having an Emb phenotype through any RNAi assay (feeding, soaking, or injecting) on WormBase. From these, in phase 1 of the screen, we identified 150 potential suppressors (plus one nonspecific suppressor; see below) of the WEE-1.3 RNAi phenotype that, when co-depleted, resulted in restoration of fertility. The suppressors differed in their ability to suppress the WEE-1.3 RNAi-induced infertility and were classified according to the level of suppression as none, weak, moderate, or strong (Figure 4A). Of the clones tested, 1723 (92%) failed to suppress, 94 (5%) weakly suppressed, 47 (2.5%) moderately suppressed, and 10 (0.5%) strongly suppressed the infertility phenotype. An entire list of suppressing clones can be found in Table S2. All of the suppressing clones were sequenced to verify the RNAi clone tested (Table S2).

To determine whether the candidate suppressors exhibit specific suppression of *wee-1.3* or are global suppressors of RNAi, we asked if any could suppress the embryonic lethality phenotype associated with *lit-1* RNAi treatment. Only one clone suppressed both the *wee-1.3* and *lit-1* RNAi depletion phenotypes (data not shown). That gene was *sago-2*, which encodes an Argonaute homolog that is partially required for the amplification phase of the RNAi response (Table 2 and Table S2) (Yigit *et al.* 2006). All of the remaining 150 suppressors were specific for *wee-1.3(RNAi)*.

**Table 2:** The 44 identified suppressors in the WEE-1.3 RNAi screen

Gene	Average Brood Size*^a^*	Average Hatching %*^a^*	Predicted Function
sago-2*^b^*	153.9	95.50	Argonaute homolog partially required for the amplification phase of RNAi responses
cdk-7	136.2	6.66	Cyclin-dependent kinase
cdk-1	132.8	1.69	Cyclin-dependent kinase
snr-1	86.5	11.60	Ortholog of human small nuclear ribonucleoprotein SmD3, biogenesis and function of snRNPs
Y49E10.21	65.7	38.21	Novel protein of unknown function
B0336.7	64.8	30.16	Protein with a THAP or THAP-like domain
rpn-9	51.6	3.85	Non-ATPase subunit of the 19S regulatory complex of the proteasome
rpn-8	32.5	2.32	26S proteasome regulatory complex, subunit RPN8/PSMD7
unc-97	32.4	33.56	LIM domain-containing protein of the PINCH family, highly similar to human LIMS1 and LIMS2
attf-2	30.2	27.29	AT hook Transcription Factor family
R10H10.3	26.9	31.13	C-type lectin
msh-6	25.8	22.71	Mismatch repair ATPase MSH6
rpt-5	25.4	7.92	Triple A ATPase subunit of 26S proteasome’s 19S regulatory particle (RP) base subcomplex
bir-2	22.7	30.81	Protein with two BIR domains that may be involved in apoptosis
rpn-2	21.4	4.65	Non-ATPase subunit of the 26S proteasome’s 19S regulatory particle (RP) base subcomplex
etr-1	21.3	19.15	Muscle-specific ELAV-type RNA-binding protein
rpn-6.1	19.9	0.75	Non-ATPase subunit of the 19S regulatory complex of the proteasome
F36A2.7	18.5	27.28	Novel protein of unknown function
acdh-12	17.8	17.34	Orthologous to the human gene ACYL-CoA DEHYDROGENASE, VERY LONG-CHAIN
acr-23	17.2	25.38	Subunit of the nicotinic acetylcholine receptor which encode ligand-gated ion channels
F11A3.2	15.6	31.03	Translation initiation factor, guanine nucleotide exchange factor
C18E3.9	15.4	16.80	Novel protein of unknown function
cul-2	15.0	6.02	E3 ubiquitin ligase
prx-5	14.3	15.18	Ortholog of the human receptor for type I peroxisomal targeting signal protein, PXR1 (or PEX5)
mop-25.3	12.3	24.28	Divergent ortholog of fission yeast Mo25p and budding yeast Hym1p
C47E8.4	12.3	21.08	Conserved protein that is a member of the FAM50A/XAP5 family of proteins
K04H4.2	11.9	25.69	Secreted protein with an N-terminal chitin-binding peritrophin-A domain
C14B9.2	11.3	11.77	Protein disulfide isomerase (prolyl 4-hydroxylase beta subunit)
unc-11	11.1	25.70	Clathrin-adaptor protein that functions in clathrin-mediated endocytosis
taf-1	10.4	9.75	Transcription initiation factor
B0280.9	10.3	13.91	Novel protein of unknown function
sel-8	10.2	18.25	Nuclear protein required for GLP-1 and LIN-12 signaling
imb-2	10.1	11.81	Importin beta family protein
grk-1	9.1	23.40	Serine/threonine protein kinase, most closely related to G protein-coupled receptor kinases
inx-14	8.6	10.10	Member of the innexin family
F42H10.5	7.9	15.39	Novel protein of unknown function
cogc-2	6.8	11.37	Ortholog of mammalian COG-2, a subunit of lobe A of the conserved oligomeric Golgi complex
sex-1	6.4	16.52	DNA-binding protein, nuclear hormone receptor superfamily of transcriptional regulators
F02A9.4	5.8	25.10	Orthologous to human SIMILAR TO METHYLCROTONOYL-COENZYME A CARBOXYLASE 2 (BETA)
henn-1	5.7	30.06	HEN1 ortholog, RNA 3′ end methyltransferase
sdz-24	4.8	13.17	*SKN-1 Dependent Zygotic transcript*, single-stranded DNA-binding replication protein A
glr-6	4.5	9.62	Putative non-NMDA ionotropic glutamate receptor subunit
mrps-30	4.4	7.88	Mitochondrial 28S ribosomal protein S30
C36E8.1	4.2	16.55	Novel protein of unknown function
nud-1	3.3	11.49	Nuclear distribution protein

a reported averages for animals co-depleted of WEE-1.3 and the indicated candidate gene

b sago-2 is a global RNAi suppressor that acts by reducing RNAi efficacy and therefore is not considered a true suppressor.

Co-depletion of CDK-1 and WEE-1.3 has been shown to suppress the infertility of WEE-1.3 depletion alone (Burrows *et al.* 2006). Our screen, which was performed blindly, successfully identified this known suppressor in three separate tests (the *cdk-1* feeding construct was present in the library as three separate clones). Furthermore, the degree of suppression was reproducible, as CDK-1 was classified as a moderate suppressor in each instance (Table S2).

In phase 2 of the screen, we sought to quantify the degree of suppression exhibited by the moderate and strong candidate suppressors (n = 51) and determine the average brood size upon suppression of the WEE-1.3 depletion infertility (Figure 4B). Only 51 of the total 57 moderate and strong suppressors were tested because the remaining six either represented duplicate RNAi clones in the library or repeatedly failed to grow during phase 2 of the screen. Quantification allowed us to identify two different classes of suppressors: those that suppressed to a wild-type level of fertility (n = 3, red bars, Figure 4B) and those that suppressed the infertility phenotype associated with WEE-1.3 depletion but did not restore fertility to a wild-type level (n = 48, gray bars, Figure 4B). Six of the 51 potential candidate suppressors were determined to have no significant difference in brood size of animals co-depleted of *wee-1.3* and the control gene, and one candidate, *sago-2*, was determined to be a global RNAi suppressor, as mentioned above (Figure 4B).

This analysis provided an additional way to categorize the suppression data by determining the percent hatching exhibited by animals co-depleted of WEE-1.3 and a candidate suppressor. Our initial expectation was that if the sterility was suppressed, then complete embryonic lethality would result because we know that *wee-1.3* mutants are embryonic lethal and all the candidates tested were also reported to be embryonic lethal. However, for many of the candidates tested in phase 2, a portion of the laid embryos successfully hatched into larvae (Table 2). The percent hatching was between 2% and 40% for all candidates, with the sole exception being *sago-2* with 95% hatching. Twenty-seven out of the 44 suppressors (61%) exhibited hatching percentages less than 20% (Table 2).

Through the two phases of the RNAi screen, we identified 44 potential suppressors of the WEE-1.3 depletion infertility, of which 43 were novel suppressors (Table 2). The one exception was *cdk-1*, which had been previously shown to suppress *wee-1.3(RNAi)*.

### GO analysis of potential suppressors

To identify functional themes among the *wee-1.3 RNAi* suppressors, we searched for enriched gene ontology (GO) annotations (Figure S2) using the DAVID bioinformatics comparison tool (Huang *et al.* 2009a,b). This strategy allowed us to identify sub-ontologies within both the biological process (BP) and cellular component (CC) gene ontology categories that were specifically enriched in our dataset of candidate suppressors. Enrichment values within the BP category were for genes involved in tissue morphogenesis, meiosis/mitosis, nuclear migration, and RNA processing. Within the CC category, enrichment values were for intracellular organelle/nucleus and proteasome complex.

### Co-depletion of CDK-7 and WEE-1.3 suppresses the WEE-1.3 depletion phenotype

**Figure 5:**
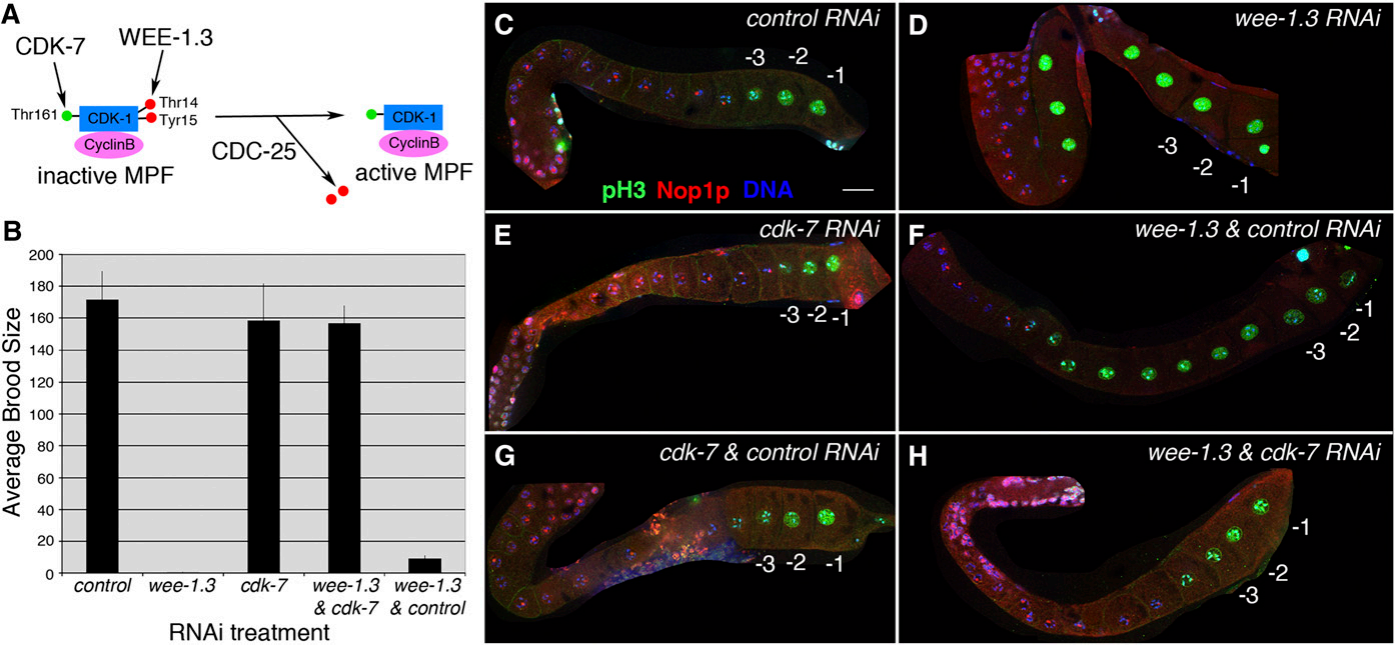
Co-depletion of WEE-1.3 and CDK-7 suppresses the infertility phenotype of WEE-1.3 depletion. (A) Schematic on how CDK-7 (CAK or Cdk-activating kinase) and WEE-1.3 (inhibitory kinase) act on maturation promoting factor (MPF) to regulate the activity of MPF. (B) Average brood size per mother treated with *control* RNAi, *wee-1.3* RNAi, *cdk-7* RNAi, or co-depleted of *wee-1.3* and *cdk-7* RNAi or *wee-1.3* and *control* RNAi. Each bar represents the average of four independent experiments with three animals per experiment (n = 12), and the error bars represent SEM. (C–H) Single-plane confocal images of gonads dissected from mothers treated with the indicated RNAi, fixed, and co-stained with antibodies against phosphohistone H3 (Ser10) (pH3, green), nucleolus (Nop1p, red), and DNA (DAPI, blue). RNAi treatment is as follows: (C) control; (D) WEE-1.3–depleted; (E) CDK-7–depleted; (F) co-depletion of WEE-1.3 and control; (G) co-depletion of CDK-7 and control; and (H) co-depletion of WEE-1.3 and CDK-7. Gonads are oriented with the proximal region to the right in this figure. Scale bar is approximately 20 μm. Individual panels of Nop1p and pH3 antibody staining can be found in Figure S3.

CDK-7 has been shown to function in *C. elegans* as a CDK-activating kinase (CAK) essential for cell-cycle progression (Figure 5A) (Wallenfang and Seydoux 2002). This kinase phosphorylates a key residue on CDK-1, the CDK component of maturation promoting factor (MPF), which is required to make a functional, active MPF. Severe loss of *cdk-7* activity, accomplished through *cdk-7(RNAi)* feeding to animals homozygous for the mutant allele *cdk-7(ax224)*, led to a one-cell embryonic arrest phenotype, similar to CDK-1 loss-of-function (Burrows *et al.* 2006; Wallenfang and Seydoux 2002). In our suppressor screen, CDK-7 was identified as a moderate suppressor of the WEE-1.3 depletion infertility phenotype (Table S2).

To show this quantitatively, we conducted brood size analysis using animals fed RNAi to a control gene, *wee-1.3* alone, *cdk-7* alone, *wee-1.3* plus *cdk-7*, or *wee-1.3* plus the control gene (Figure 5B). For these experiments, we define a brood as the total number of embryos laid and larvae hatched on a given plate. The average brood size for animals fed *control(RNAi)* was 172 compared with 0.6 for animals fed *wee-1.3(RNAi)*. *cdk-7(RNAi)* by itself results in an average brood size of 158; however, that entire brood is comprised of multi-cellular arrested embryos compared with the broods of viable progeny observed on *control(RNAi)* treatment (Figure 5B). Co-depletion of WEE-1.3 and CDK-7 via RNAi feeding returns the brood size to a wild-type level (average brood = 157), and the entire brood consists of multi-cellular embryos (Figure 5B). In contrast, co-depletion of WEE-1.3 and a control gene resulted in an average brood of nine viable progeny (Figure 5B).

We then examined the gonads of animals co-depleted of WEE-1.3 and CDK-7 by immunofluorescence using antibodies to known oocyte maturation markers (Burrows *et al.* 2006) to determine if the gonads exhibited precociously matured oocytes (Figure 5, C–H; individual panels of antibody staining can be found in Figure S3). Maturing oocytes exhibit a condensed chromatin state that can be monitored by staining with an antibody specific for phosphorylated histone H3 at serine 10 (pH3) (Hendzel *et al.* 1997; Hsu *et al.* 2000). In addition, mature oocytes lack a nucleolus, and the absence or presence of a nucleolus can be visualized by using an antibody to the nucleolar marker Nop1p/fibrillarin (Aris and Blobel 1988; Henriquez *et al.* 1990; Schimmang *et al.* 1989). Animals fed RNAi to a control gene exhibited a wild-type pattern of pH3 and Nop1p expression. Nop1p staining was absent in the nuclei of the −1 and −2 oocytes, whereas the pH3 antibody stained the nuclei and chromosomes of the three most proximal oocytes within each gonad arm (Figure 5C). As previously reported, *wee-1.3(RNAi)* animals contain precociously maturing oocytes when compared with *control(RNAi)* animals (Burrows *et al.* 2006). This is characterized by an increased number of pH3–positive oocyte nuclei and a decreased number of Nop1p-positive oocyte nuclei (Figure 5D). Depletion of CDK-7 via RNAi feeding did not alter the expression pattern of either pH3 or Nop1p when compared with *control(RNAi)* animals (Figure 5E). Co-depletion of both WEE-1.3 and CDK-7 suppressed the precocious oocyte maturation phenotype exhibited by depletion of WEE-1.3 alone and returned the gonad to a more wild-type expression pattern, with the three to four most proximal oocytes being pH3-positive and Nop1p-negative (Figure 5H). Co-depletion of either WEE-1.3 or CDK-7 in combination with a control protein did not change the expression pattern of pH3 and Nop1p within the gonads when compared with single depletion of WEE-1.3 or CDK-7 (Figure 5F-G). Therefore, suppression of the precocious oocyte maturation caused by *wee-1.3(RNAi)* is specific to *cdk-7(RNAi)*.

## Discussion

In this study, we further characterized the precocious oocyte maturation observed in WEE-1.3–depleted animals and identified a number of interactors with WEE-1.3 through a large-scale RNAi suppressor screen. Our findings suggest that upon WEE-1.3 depletion by RNAi, the oocytes of the treated animals prematurely relocalize proteins to resemble the pattern frequently observed during early embryogenesis. These oocytes also undergo precocious embryonic gene activation. The suppressor screen successfully identified 44 potential interactors with WEE-1.3, which are targets for future analysis.

The expression of WEE-1.3 in the proximal germline within developing oocytes supports a role for WEE-1.3 in oocyte maturation. In addition, the ubiquitous expression of WEE-1.3 throughout multiple tissues types, including the soma of the adult worm and the developing embryo, suggests that WEE-1.3 may have previously unidentified roles in the somatic tissues, presumably to regulate other processes besides oocyte maturation. Supporting the idea of zygotic roles for WEE-1.3 in embryonic development are unpublished observations by our laboratory that null mutants of *wee-1.3(ok729)* can give rise to homozygous progeny that are predominantly late embryonic lethal and rare L1 larval lethal animals.

WEE-1.3 is predicted to be a transmembrane protein, and our results show that WEE-1.3 is localized to a perinuclear position in both germline and somatic tissues. This localization fits nicely with the current known role for WEE-1.3, because this protein has been shown to interact with CDK-1, and CDK-1 is a protein that shuttles between the cytoplasm and the nucleus to regulate its target genes. Having WEE-1.3 positioned on the nuclear periphery places that protein in the ideal location to regulate CDK-1. Meanwhile, the diffuse cytoplasmic punctate expression of WEE-1.3 and co-localization with known endoplasmic reticulum markers suggests that there might be other proteins with which WEE-1.3 interacts and regulates. Our described localization pattern is similar to those reported for orthologs of the Myt1/Wee1 family of kinases in mouse, *Drosophila*, and humans. In mouse isolated GV oocytes, WEE1B was found throughout the nucleus, whereas Myt1 exhibited a punctate pattern throughout the cytoplasm and was excluded from the nucleus (Oh *et al.* 2010). Meanwhile, in *Drosophila* S2 cells, dMyt1 subcellular localization was shown to overlap with Golgi markers (Cornwell *et al.* 2002). Furthermore, in HeLa cells, human Myt1 was localized to the ER and Golgi complex (Liu *et al.* 1997).

The observed diakinetic chromosome coating exhibited by the WEE-1.3 antibody is extremely intriguing and provides evidence for a potential new role for this particular inhibitory kinase. In the initial studies of WEE-1.3–depleted animals, it was demonstrated that the oocyte chromosomes do not maintain a diakinetic arrangement, but rather coalesce and over-congress into a single mass of chromatin (Burrows *et al.* 2006). This implied that there was some required role for WEE-1.3 in maintenance of mature oocyte chromosome structure. Our studies help to confirm this role and provide the basis for a potential mechanism by which WEE-1.3 could be involved in this maintenance. Because WEE-1.3 was shown to coat all of the diakinetic chromosomes in wild-type animals, we propose that WEE-1.3 is necessary for the individualization of the chromosomes in the mature oocytes and, thus, in the absence of WEE-1.3, the chromosomes amalgamate to form one single structure. This is an area we are actively investigating.

### WEE-1.3 depletion causes premature relocalization of embryonic proteins and early gene activation within the precociously maturing oocytes

The initiation of development upon fertilization, whereby a quiescent oocyte transitions into a dynamic embryo capable of differentiating into multiple cell types, is marked by the relocalization or degradation of a number of maternal proteins. Some of these proteins (*e.g.*, MBK-2, EGG-3, and CHS-1) have been previously reported to exhibit premature relocalization in the oocytes of WEE-1.3–depleted animals (Burrows *et al.* 2006; Cheng *et al.* 2009; Stitzel *et al.* 2007). Our additional observations of PGL-1 and OMA-1 exhibiting premature relocalization and degradation upon WEE-1.3 depletion demonstrate that the redistribution and degradation of these two proteins are dependent on meiotic maturation and not fertilization. Alternatively, this altered localization could be the result of embryonic gene activation. The premature expression of CBD-1::mCherry in WEE-1.3–depleted oocytes provides the first indication regarding why those oocytes might be fertilization-incompetent and *wee-1.3(RNAi)* results in infertility. With the oocytes prematurely expressing a component of the eggshell, this could potentially influence the competency for fertilization in the WEE-1.3–depleted oocytes. It will be interesting to determine if other recently discovered components of the eggshell are also prematurely expressed in oocytes of WEE-1.3–depleted animals (Olson *et al.* 2012).

Early gene activation (EGA) of embryonic transcripts was also observed in WEE-1.3–depleted animals. Two of the earliest known transcribed mRNAs in *C. elegans* (*vet-1* and *vet-4*) were shown to have an increased abundance in the germline upon *wee-1.3(RNAi)* treatment. Because the germlines were normalized to control RNAi-treated germlines, and because the only difference was the presence of precociously maturing oocytes, the data imply that the normally quiescent oocytes have induced embryonic-like transcription. The fact that not all transcripts are prematurely activated (*e.g.*, *vet-6* mRNA) is not surprising due to the fact that we showed that while the OTE transition has occurred prematurely with respect to a number of proteins (*e.g.*, MBK-2 and CHS-1), complete OTE has not occurred. Namely, we did not observe premature relocalization of CAV-1 in the precociously maturing oocytes, nor did we observe the formation of a chitinous eggshell. In addition, there is no reason to believe that the precocious oocytes have begun to transcribe every embryonic gene.

The connection between the meiotic cell cycle and precocious EGA has not been previously observed. However, Biedermann *et al.* (2009) recently demonstrated a link between the mitotic cell cycle and EGA in *C. elegans*. They reported that loss of GLD-1 in the germline resulted in ectopic activation of Cyclin E (CYE-1) and CDK-2 and premature onset of embryonic-like transcription or EGA. The authors proposed that the precocious EGA might be due to CYE-1/CDK-2 activating a transcription factor involved in EGA. The precise mechanism(s) by which EGA is controlled is not well-understood, but studies in different model systems have implicated degradation of maternal mRNAs, repression of basic transcription factors, and induction of gene expression through a sequence-specific transcription factor (Guven-Ozkan *et al.* 2008; Liang *et al.* 2008; Tadros *et al.* 2007). In addition, it has been shown that activation of CDK2 by maternal cyclin A2 is required for EGA in the one-cell mouse embryo (Hara *et al.* 2005). How does depletion of WEE-1.3 result in precocious EGA? We propose that the absence of WEE-1.3 might result in premature activation of CDK-1 in the oocytes. This would lead to the inappropriate activation of the target genes of CDK-1 in the oocytes. Normally, CDK-1 would be held inactive in the oocytes, and the target genes only activated in the fertilized embryos.

### Identification of suppressors of the WEE-1.3 depletion phenotype through an RNAi screen

The RNAi suppressor screen allowed us to identify many genes that resulted in a restoration of fertility when co-depleted with WEE-1.3. The mechanism of suppression remains to be determined and is a focus of future investigation. In total, we identified 44 potential interactors with WEE-1.3 that had not been previously predicted or shown to physically interact. Importantly, we had more than 1600 internal controls that did not suppress the WEE-1.3 depletion infertility when co-depleted along with *wee-1.3*, suggesting that the effects we see after phase 2 of the screen are real and not due to simple dilution effects. In addition, we performed dilution analysis of the effectiveness of co-diluted *wee-1.3(RNAi)* with a control RNAi that does not affect fertility (*smd-1*), and we found that diluting *wee-1.3* by five-fold does not alter the observed infertility phenotype (data not shown).

Interestingly, of the 38 predicted and/or confirmed interactions identified on WormBase for WEE-1.3, we tested 16 (42%) in our screen and found 3 (8%) that acted as suppressors of WEE-1.3 depletion infertility. In addition, one of the suppressors, IMB-2, had been shown to interact via two-hybrid studies by Boxem *et al.* (2008), and our data provide support that this interaction also occurs *in vivo*. We note that, because these candidates were identified through a genetic screen, it is possible that many are functioning indirectly with WEE-1.3 to cause suppression of the infertility phenotype.

We initially chose to screen only the RNAi library clones that had been previously characterized as embryonic lethals on the assumption that any WEE-1.3 interactors would be essential genes. Interestingly, of the 1874 Emb clones we screened through feeding, a number of the suppressors were not embryonic lethal by RNAi feeding, yet they suppressed the WEE-1.3 depletion infertility. This includes most of the strong suppressors. The fact that these clones were not Emb in our experiments can be explained by the fact that, as previously noted, the genes screened were ones reported as having an Emb phenotype in WormBase through any RNAi assay (feeding, injecting, soaking, feeding in a sensitized strain background). Thus, a stronger method of RNAi other than feeding may be required to observe the Emb phenotype of that gene. In addition, this implies that a genome-wide RNAi screen might identify even more candidate suppressors.

The bioinformatics analysis of gene ontologies conducted on our list of suppressors enabled us to extract functionally relevant biology from the large gene list. Although many suppressors were cell cycle–related, a number failed to fall into that category; thus, this screen has potentially identified non-cell cycle functions of WEE-1.3 or potentially has identified genes that have previously unidentified roles in the cell cycle. A number of components of the proteasome were identified as suppressing the *wee-1.3(RNAi)* infertility phenotype, indicating the importance that appropriate protein degradation has in the germline, especially in oocyte maturation.

Because RNAi provides a very unique window of protein depletion, which may or may not result in a complete lack of the targeted gene product, our screen potentially identified many suppressor candidates that would have been difficult to identify if screening a mutant collection for suppression. We have begun to confirm whether genetic mutants of identified suppressors also suppress the WEE-1.3 depletion phenotype. Preliminary data indicate that some, but not all, mutants can suppress the *wee-1.3(RNAi)* infertility to a level similar to that observed upon RNAi depletion of the candidate (data not shown).

Finally, it is worth pointing out that the suppressors identified in this screen might potentially be acting as direct regulators of CDK-1. For example, suppressors that alter the trafficking or localization of CDK-1 could result in suppression of the WEE-1.3 depletion infertility. We hypothesize that if CDK-1 is prevented from translocating to the nucleus, then the CDK-1 target genes would no longer be erroneously activated, and this might prevent the precociously maturing oocytes. The suppressor IMB-2, a member of the importin beta family of proteins that bind and transport proteins into the nucleus, might be functioning in this manner (Adam 2009). Co-depletion of WEE-1.3 and IMB-2 could potentially result in precociously activated CDK-1; however, due to the absence of IMB-2, the activated CDK-1 cannot translocate to the nucleus.

The suppressor CDK-7 (Figure 5) is most likely suppressing through its action on CDK-1 and not directly by interacting with WEE-1.3. CDK-7 is the CDK activating kinase (CAK) involved in positively phosphorylating the CDK component of maturation promoting factor (MPF). This activating phosphorylation is essential for the proper activity level of MPF. By depleting both CDK-7 and WEE-1.3, the level of active MPF might be modulated such that there is enough functionally active MPF to alleviate meiotic arrest and continue with the meiotic cell cycle.

In summary, our studies have determined the *in vivo* distribution of WEE-1.3, the *C. elegans* Wee1/Myt1 ortholog, and further characterized the WEE-1.3 depletion phenotype. We have shown that the precocious oocyte maturation observed upon WEE-1.3 depletion results not only in the premature relocalization of maternal proteins but also in the premature appearance of an eggshell component and in embryonic gene activation. Finally, we have identified a large number of potential interactors and/or regulators of WEE-1.3 through an RNAi suppressor screen. This screen has generated a rich resource for future inquiry into the studies of cell cycle regulation and, specifically, oocyte maturation.

## Supplementary Material

Supporting Information
