# Supplementary material for: An RNAi-Based Suppressor Screen Identifies Interactors of the Myt1 Ortholog of Caenorhabditis elegans
Source: G3 (Bethesda). 2014 Oct 8;4(12):2329–43. doi: 10.1534/g3.114.013649 (PMC4267929; doi:10.1534/g3.114.013649)
Supplement: Supporting Information [file supp_4_12_2329__index.html]

An RNAi-Based Suppressor Screen Identifies Interactors of the Myt1 Ortholog of Caenorhabditis elegans — Supporting Information 

# An RNAi-Based Suppressor Screen Identifies Interactors of the Myt1 Ortholog of *Caenorhabditis elegans*

## Supporting Information for Allen, Nesmith, and Golden, 2014

**Files in this Data Supplement:**

- Supporting Information - Figures S1-S3 and Tables S1-S2 (PDF, 415 KB)
- Figure S1 - WEE-1.3 is localized to the nuclear envelope and a portion of the endoplasmic reticulum. (PDF, 237 KB)
- Figure S2 - Gene ontology (GO) terms enriched in the set of suppressors identified in the WEE-1.3 RNAi suppressor screen. (PDF, 100 KB)
- Figure S3 - Individual panels of NOP-1 and pH3 staining for images found in Figure 5. (PDF, 212 KB)
- Table S1 - Plasmids and Primers Used in This Study (PDF, 81 KB)
- Table S2 - .xls, 643 KB
