# Supplementary material for: An RNAi-Based Suppressor Screen Identifies Interactors of the Myt1 Ortholog of Caenorhabditis elegans
Source: G3 (Bethesda). 2014 Oct 8;4(12):2329–43. doi: 10.1534/g3.114.013649 (PMC4267929; doi:10.1534/g3.114.013649)
Supplement: Supporting Information [file supp_g3.114.013649_013649SI.pdf]

G3: Genes | Genomes | Genetics

An RNAi-based suppressor screen identifies interactors of the Myt1 ortholog of *C. elegans*

Anna K. Allen[1], Jessica E. Nesmith[2], and Andy Golden

Laboratory of Biochemistry and Genetics, National Institute of Diabetes and Digestive and Kidney Diseases, National Institutes of Health, Bethesda, Maryland 20892

[1]Present address: Department of Biology, Howard University, Washington, DC 20059

[2]Present address: Biological and Biomedical Sciences Program, University of North Carolina, Chapel Hill, NC 27599

Corresponding Author:

Anna Allen

EE Just Hall, Room 244

Howard University

Washington, DC 20059

E-mail: anna.allen@howard.edu

DOI: 10.1534/g3.114.013649

FIGURE S1

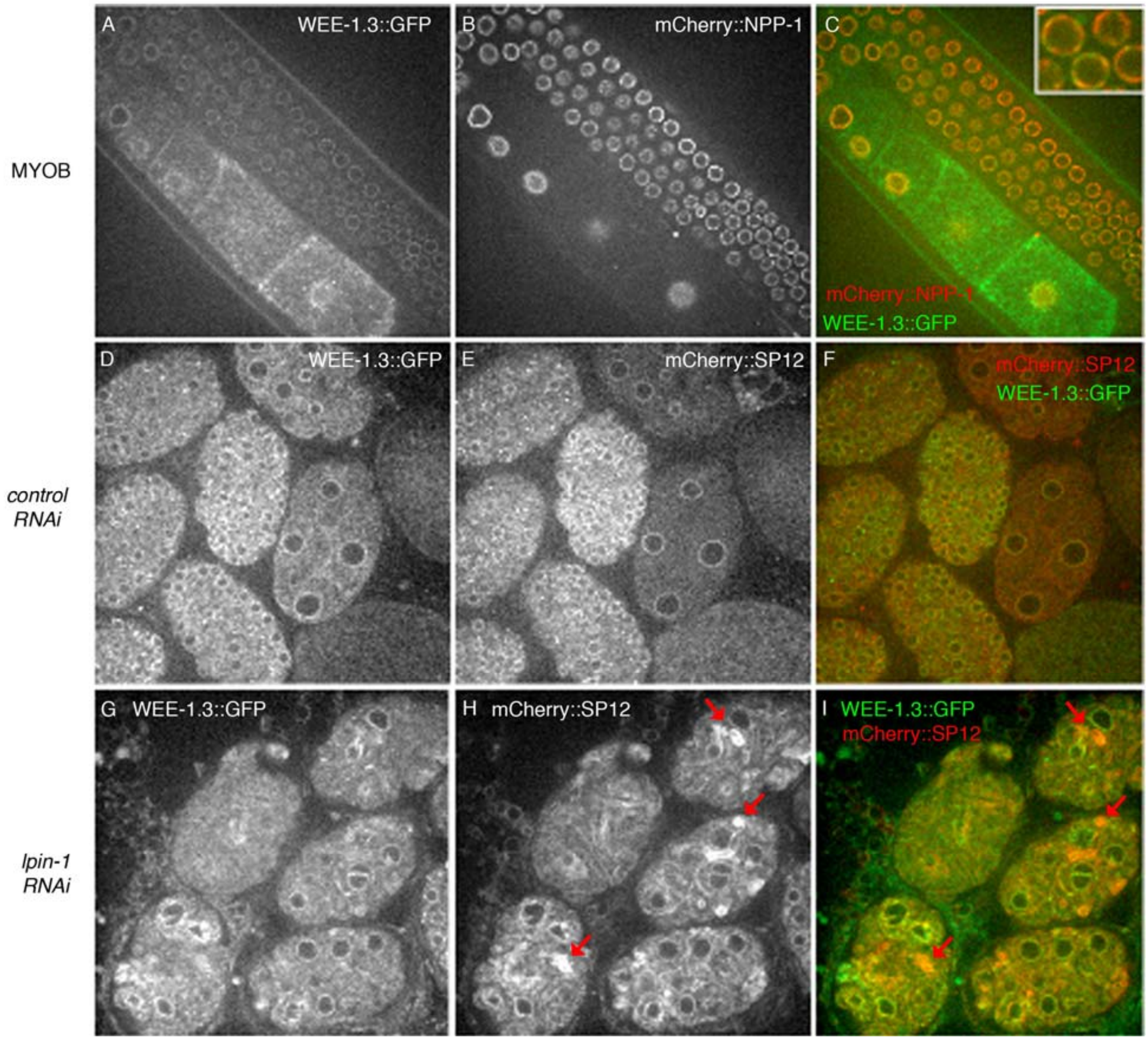

Figure S1 WEE-1.3 is localized to the nuclear envelope and a portion of the endoplasmic reticulum. Single confocal images of live animals expressing WEE-1.3::GFP (green) and mCherry::NPP-1 (red) (A-C) or WEE-1.3::GFP (green) and mCherry::SP12 (red) (D-I). (A-C) Gonad from animal expressing WEE-1.3::GFP and mCherry::NPP-1. Inset in (C) is a blow-up of meiotic nuclei in the more distal germline. (D-I) Embryos from animals expressing WEE-1.3::GFP and mCherry::SP12 subjected to *control* (D-F) or *lpin-1* (G-I) RNAi. Red arrows in (H) and (I) indicate regions where WEE-1.3::GFP and mCherry::SP12 do not colocalize. Embryos are ~50μm in length.

Figure S2

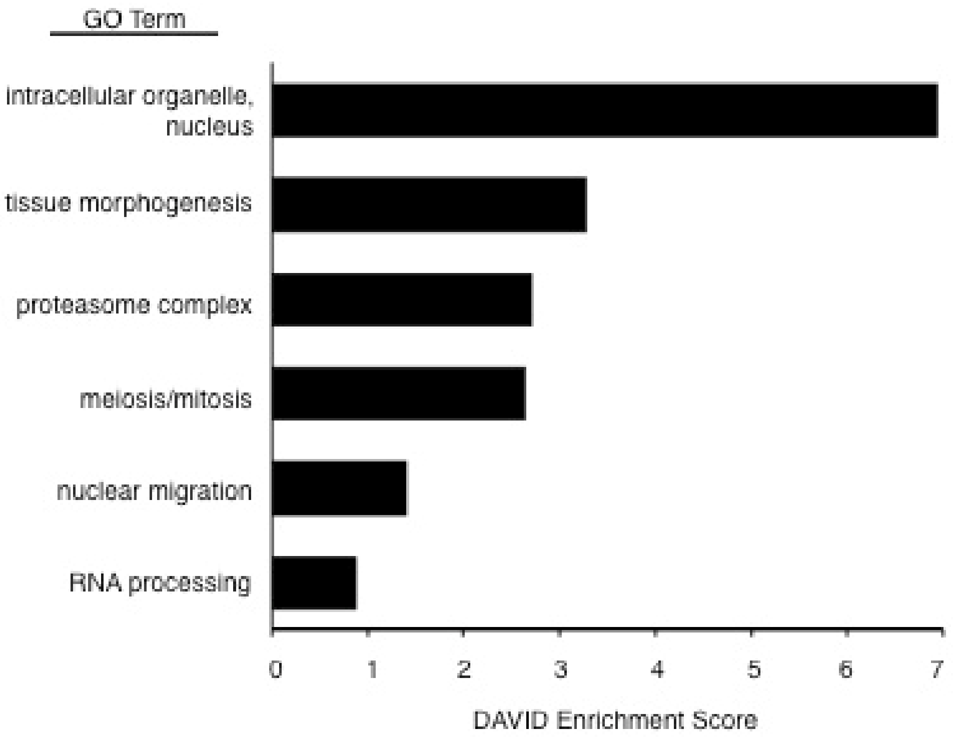

Figure S2 Gene ontology (GO) terms enriched in the set of suppressors identified in the WEE-1.3 RNAi suppressor screen. The top scoring GO: Biological Process and GO: Cellular Component categories with *P* values of <0.05 are shown. Reported terms are subontologies that belong to either biological process (BP; tissue morphogenesis, meiosis/mitosis, phosphorylation, translation, nuclear migration, RNA processing, and proteolysis) or cellular component (CC; intracellular organelle/nucleus and proteasome complex) categories. Enrichment and *P*-values are as follows: intracellular organelle/nucleus (E = 6.92, *P*-value = 9.3e-10), tissue morphogenesis (E = 3.25, *P*-value = 4.4e-4), proteasome complex (E = 2.68, *P*-value = 1.6e-4), meiosis/mitosis (E = 2.62, *P*-value = 1.0e-3), nuclear migration (E = 1.38, *P*-value = 4.3e-2), and RNA processing (E = 0.85, *P*-value = 1.8e-2).

FIGURE S3

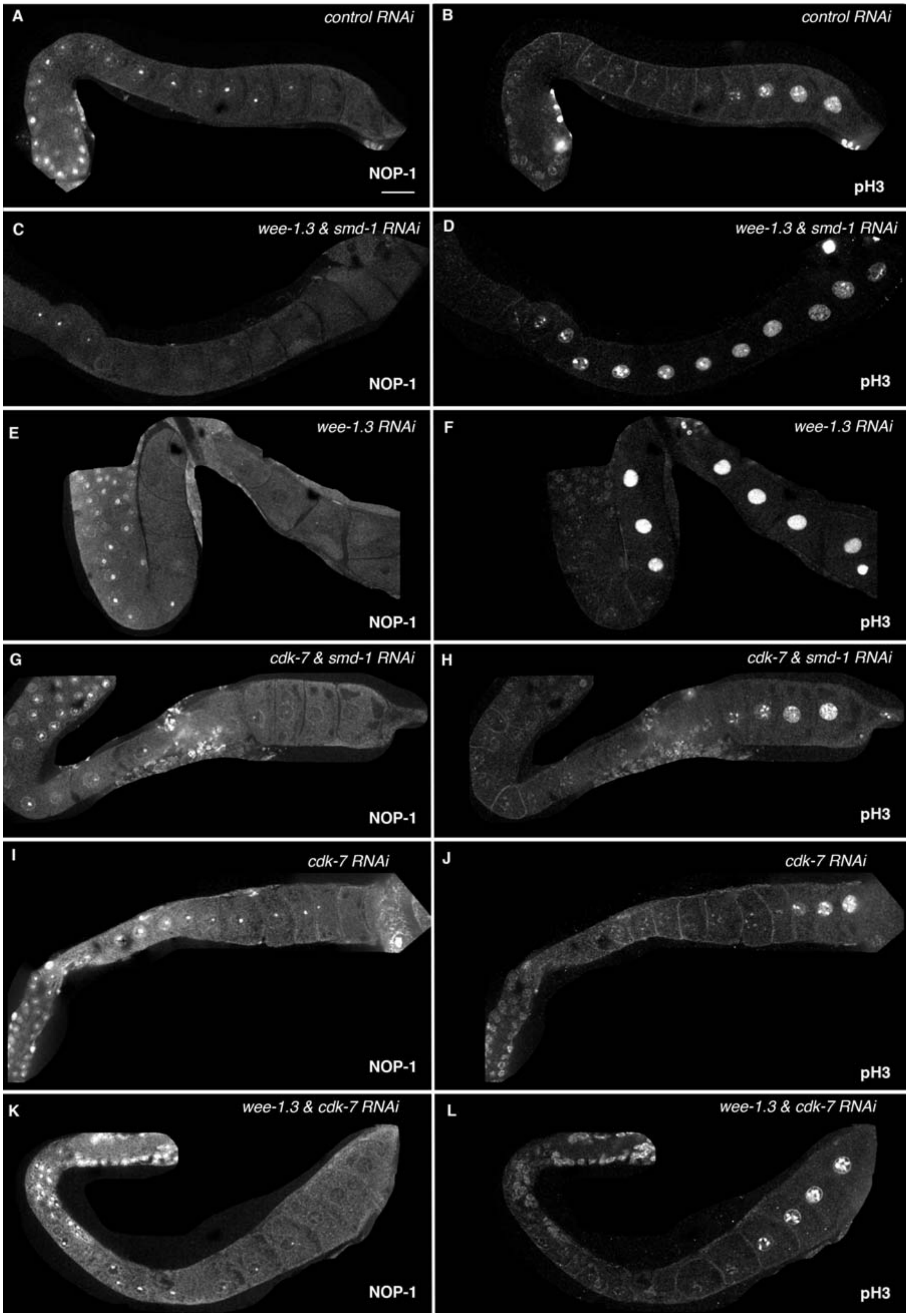
A
control RNAi
NOP-1
B
control RNAi
pH3
C
wee-1.3 & smd-1 RNAi
NOP-1
D
wee-1.3 & smd-1 RNAi
pH3
E
wee-1.3 RNAi
NOP-1
F
wee-1.3 RNAi
pH3
G
cdk-7 & smd-1 RNAi
NOP-1
H
cdk-7 & smd-1 RNAi
pH3
I
cdk-7 RNAi
NOP-1
J
cdk-7 RNAi
pH3
K
wee-1.3 & cdk-7 RNAi
NOP-1
L
wee-1.3 & cdk-7 RNAi
pH3

Figure S3 Individual panels of NOP-1 and pH3 staining for images found in Figure 5.

Individual panels for NOP-1 and pH3 antibody images provided in Figure 5. Single-plane confocal images of gonads dissected from mothers treated with the indicated RNAi, fixed, and co-stained with antibodies against the nucleolus (NOP-1; panels A, C, E, G, I, and K) and phosphohistone H3 (Ser10) (pH3; panels B, D, F, H, J, and L). RNAi treatment is as follows: (A-B) control, (C-D) co-depletion of WEE-1.3 and control, (E-F) WEE-1.3-depleted, (G-H) co-depletion of CDK-7 and control, (I-J) CDK-7-depleted, and (K-L) co-depletion of WEE-1.3 and CDK-7. Gonads are oriented with the proximal region to the right in this figure. Scale bar is approximately 20μm.

Table S1 Plasmids and Primers Used in This Study

PLASMID	DESCRIPTION OF PLASMID INSERT	PRIMERS USED TO GENERATE PLASMID	DONOR VECTOR USED
pAA11	attL4::*wee-1.3* short promoter::attR1	oAKA2 and oAKA3	pDONR(P4-P1r)
pAA13	attR2::*wee-1.3* 3'UTR::attL3	oAKA6 and oAKA9	pDONR(P2r-P3)
pAA15	attR2::*wee-1.3* gene + *wee-1.3* 3'UTR::attL3	oAKA5 and oAKA9	pDONR(P2r-P3)
pAA32	attL4::*wee-1.3* short promoter + *wee-1.3* gene::attR1	oAKA51 and oAKA52	pDONR(P4-P1r)
pCR110	attL1-GFP with introns (no stop)-attL2		
pCR347	attL1-mCherry (no stop)-attL2		
pCR319	attR4 - attR3; *C.elegans* unc-119(+)		
pNU162	*fib-1* prom::FIB-1::eGFP::*fib-1* 3' UTR + *unc-119(+)*		
pDONR P4 P1R	attP4 – attP1R		
pDONR P2R P3	attP2R – attP3		

PRIMER	PRIMER SEQUENCE (5' → 3')	AMPLIFIES
oAKA2	GGGGACAACTTTGTATAGAAAAGTTGGCcaacatcagaaattcaag	attB4- *wee-1.3* promoter (forward)
oAKA3	GGGGACTGCTTTTTTGTACAAACTTGGcattttcactgaaaatg	attB1- *wee-1.3* promoter (reverse)
oAKA5	ggggacagctttcttgtacaaagtggTTATGGACGACACAGAGGGTAACTC	attB2- *wee-1.3* gene (forward)
oAKA6	ggggacagctttcttgtacaaagtggGGTAAtaatgcacaaaaatcagaaa	attB2- *wee-1.3* 3'UTR (forward)
oAKA9	ggggacaactttgtataataaagttgCgtcctacctccagtactacaagaacagg	attB3- *wee-1.3* 3'UTR (reverse)
oAKA51	GGGGACAACTTTGTATAGAAAAGTTGGCaccccaaccattgcaaatatgtg	attB4- *wee-1.3* promoter (forward)
oAKA52	GGGGACTGCTTTTTTGTACAAACTTGCaacctcgtcgcctgatgatcc	attB1- *wee-1.3* gene (reverse)
B4F2	ggggacaactttgtatagaaaagttggcACGTGGATGAATGAGAGCAGC	attB4- *cbd-1* promoter (forward)
B1R3	ggggactgcttttttgtacaaacttggATATCCCGATTTATCATCATTGTACG	attB1- *cbd-1* gene (reverse)
B2rF2	ggggacagctttcttgtacaaagtggccTGATAAGTTGTTCTAAACACCCCC	attB2- *cbd-1* 3'UTR (forward)
B3R1	ggggacaactttgtataataaagttggGCGTCCCTTTCACATTTACAG	attB3- *cbd-1* 3'UTR (reverse)
oAKA70	ggtggttcctccggaaagaa	*act-1* (forward)
oAKA71	gctatgttccagccatccttct	*act-1* (reverse)
oAKA88	gaataatgtgatcgacgaggctcc	*wee-1.3* (forward)
oAKA89	tcttctacgtggcgattccgattg	*wee-1.3* (reverse)
oAKA80	tctcaccatcgttctctctcactg	*vet-1* (forward)
oAKA81	tcggtgggagtgaagaatgg	*vet-1* (reverse)
oAKA84	gtgcttaactttccatccgttg	*vet-4* (forward)
oAKA85	caacccagtttctctggaagtc	*vet-4* (reverse)
oAKA76	ggaagaatgttcaagcggcac	*vet-6* (forward)
oAKA77	tccgtctctgatggcttcttctc	*vet-6* (reverse)

Table S2 is available for download as an Excel file at

http://www.g3journal.org/lookup/suppl/doi:10.1534/g3.114.013649/-/DC1
