# Supplementary material for: An RNAi-Based Suppressor Screen Identifies Interactors of the Myt1 Ortholog of Caenorhabditis elegans
Source: G3 (Bethesda). 2014 Oct 8;4(12):2329–43. doi: 10.1534/g3.114.013649 (PMC4267929; doi:10.1534/g3.114.013649)
Supplement: Supporting Information [file supp_g3.114.013649_FigureS1.pdf]

FIGURE S1

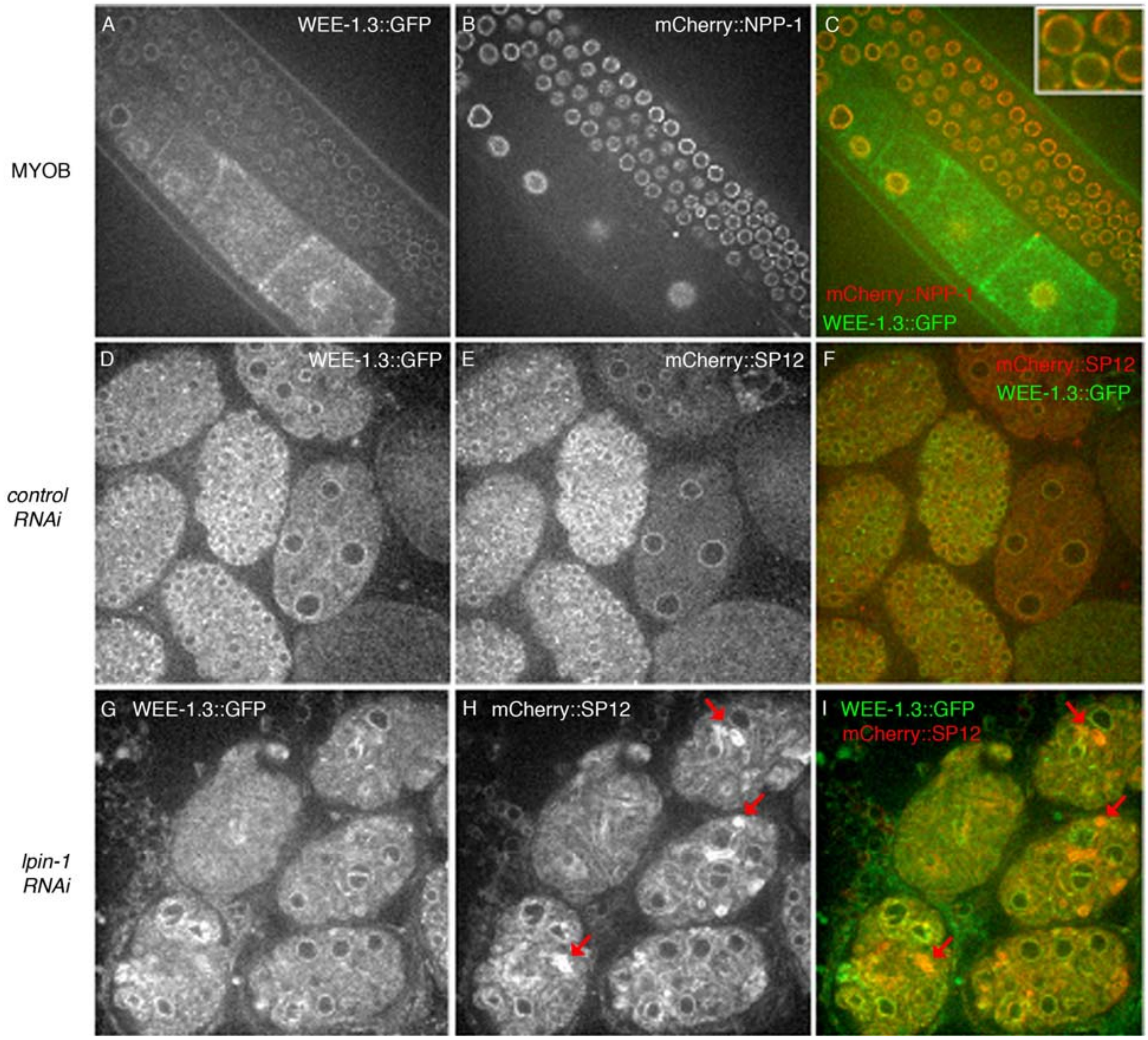

Figure S1 WEE-1.3 is localized to the nuclear envelope and a portion of the endoplasmic reticulum. Single confocal images of live animals expressing WEE-1.3::GFP (green) and mCherry::NPP-1 (red) (A-C) or WEE-1.3::GFP (green) and mCherry::SP12 (red) (D-I). (A-C) Gonad from animal expressing WEE-1.3::GFP and mCherry::NPP-1. Inset in (C) is a blow-up of meiotic nuclei in the more distal germline. (D-I) Embryos from animals expressing WEE-1.3::GFP and mCherry::SP12 subjected to *control* (D-F) or *lpin-1* (G-I) RNAi. Red arrows in (H) and (I) indicate regions where WEE-1.3::GFP and mCherry::SP12 do not colocalize. Embryos are ~50μm in length.
