# Supplementary material for: An RNAi-Based Suppressor Screen Identifies Interactors of the Myt1 Ortholog of Caenorhabditis elegans
Source: G3 (Bethesda). 2014 Oct 8;4(12):2329–43. doi: 10.1534/g3.114.013649 (PMC4267929; doi:10.1534/g3.114.013649)
Supplement: Supporting Information [file supp_g3.114.013649_FigureS2.pdf]

Figure S2

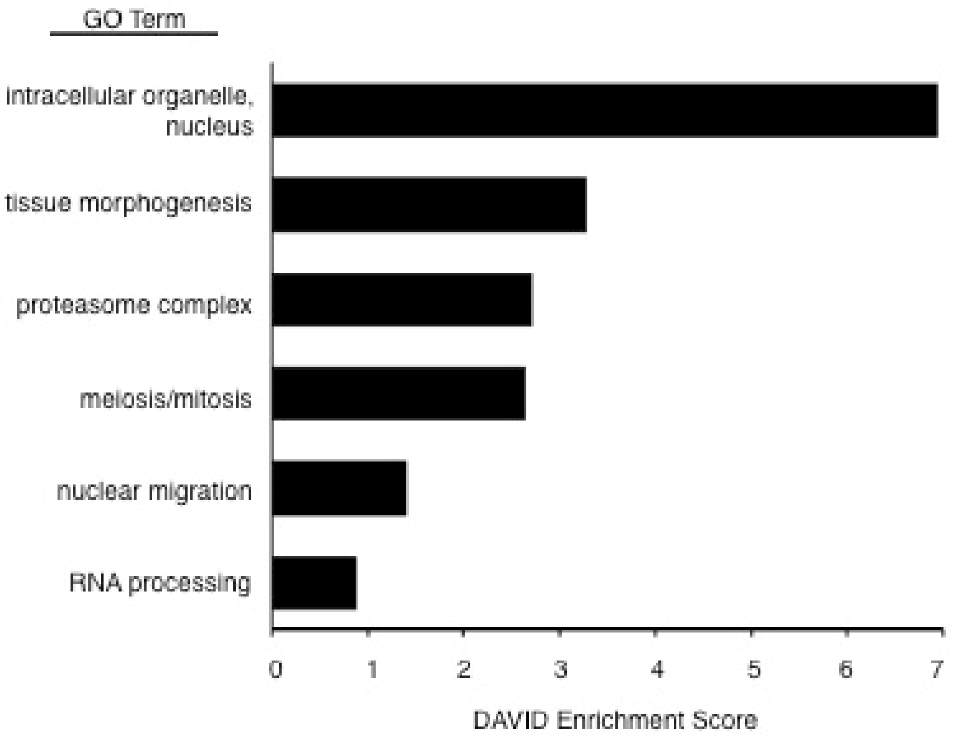

Figure S2 Gene ontology (GO) terms enriched in the set of suppressors identified in the WEE-1.3 RNAi suppressor screen. The top scoring GO: Biological Process and GO: Cellular Component categories with *P* values of <0.05 are shown. Reported terms are subontologies that belong to either biological process (BP; tissue morphogenesis, meiosis/mitosis, phosphorylation, translation, nuclear migration, RNA processing, and proteolysis) or cellular component (CC; intracellular organelle/nucleus and proteasome complex) categories. Enrichment and *P*-values are as follows: intracellular organelle/nucleus (E = 6.92, *P*-value = 9.3e-10), tissue morphogenesis (E = 3.25, *P*-value = 4.4e-4), proteasome complex (E = 2.68, *P*-value = 1.6e-4), meiosis/mitosis (E = 2.62, *P*-value = 1.0e-3), nuclear migration (E = 1.38, *P*-value = 4.3e-2), and RNA processing (E = 0.85, *P*-value = 1.8e-2).
