# Supplementary material for: An RNAi-Based Suppressor Screen Identifies Interactors of the Myt1 Ortholog of Caenorhabditis elegans
Source: G3 (Bethesda). 2014 Oct 8;4(12):2329–43. doi: 10.1534/g3.114.013649 (PMC4267929; doi:10.1534/g3.114.013649)
Supplement: Supporting Information [file supp_g3.114.013649_FigureS3.pdf]

FIGURE S3

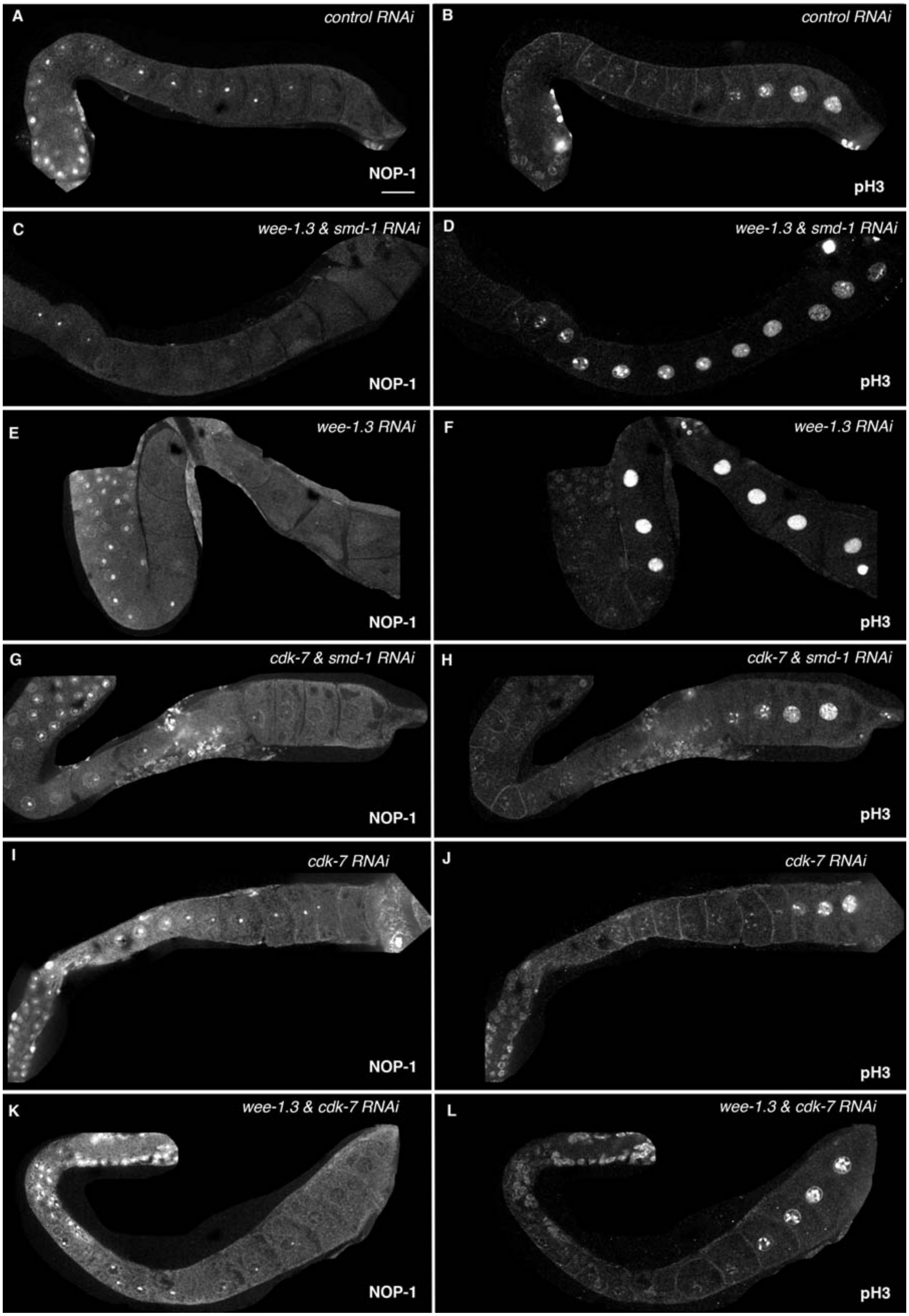
A
control RNAi
NOP-1
B
control RNAi
pH3
C
wee-1.3 & smd-1 RNAi
NOP-1
D
wee-1.3 & smd-1 RNAi
pH3
E
wee-1.3 RNAi
NOP-1
F
wee-1.3 RNAi
pH3
G
cdk-7 & smd-1 RNAi
NOP-1
H
cdk-7 & smd-1 RNAi
pH3
I
cdk-7 RNAi
NOP-1
J
cdk-7 RNAi
pH3
K
wee-1.3 & cdk-7 RNAi
NOP-1
L
wee-1.3 & cdk-7 RNAi
pH3

Figure S3   Individual panels of NOP-1 and pH3 staining for images found in Figure 5.

Individual panels for NOP-1 and pH3 antibody images provided in Figure 5.   Single-plane confocal images of gonads dissected from mothers treated with the indicated RNAi, fixed, and co-stained with antibodies against the nucleolus (NOP-1; panels A, C, E, G, I, and K) and phosphohistone H3 (Ser10) (pH3; panels B, D, F, H, J, and L). RNAi treatment is as follows: (A-B) control, (C-D) co-depletion of WEE-1.3 and control, (E-F) WEE-1.3-depleted, (G-H) co-depletion of CDK-7 and control, (I-J) CDK-7-depleted, and (K-L) co-depletion of WEE-1.3 and CDK-7. Gonads are oriented with the proximal region to the right in this figure. Scale bar is approximately 20□m.
