# Supplementary material for: An RNAi-Based Suppressor Screen Identifies Interactors of the Myt1 Ortholog of Caenorhabditis elegans
Source: G3 (Bethesda). 2014 Oct 8;4(12):2329–43. doi: 10.1534/g3.114.013649 (PMC4267929; doi:10.1534/g3.114.013649)
Supplement: Supporting Information [file supp_g3.114.013649_TableS1.pdf]

Table S1 Plasmids and Primers Used in This Study

PLASMID	DESCRIPTION OF PLASMID INSERT	PRIMERS USED TO GENERATE PLASMID	DONOR VECTOR USED
pAA11	attL4::*wee-1.3* short promoter::attR1	oAKA2 and oAKA3	pDONR(P4-P1r)
pAA13	attR2::*wee-1.3* 3'UTR::attL3	oAKA6 and oAKA9	pDONR(P2r-P3)
pAA15	attR2::*wee-1.3* gene + *wee-1.3* 3'UTR::attL3	oAKA5 and oAKA9	pDONR(P2r-P3)
pAA32	attL4::*wee-1.3* short promoter + *wee-1.3* gene::attR1	oAKA51 and oAKA52	pDONR(P4-P1r)
pCR110	attL1-GFP with introns (no stop)-attL2		
pCR347	attL1-mCherry (no stop)-attL2		
pCR319	attR4 - attR3; *C.elegans* unc-119(+)		
pNU162	*fib-1* prom::FIB-1::eGFP::*fib-1* 3' UTR + *unc-119(+)*		
pDONR P4 P1R	attP4 – attP1R		
pDONR P2R P3	attP2R – attP3		

PRIMER	PRIMER SEQUENCE (5' → 3')	AMPLIFIES
oAKA2	GGGGACAACTTTGTATAGAAAAGTTGGCcaacatcagaaattcaag	attB4- *wee-1.3* promoter (forward)
oAKA3	GGGGACTGCTTTTTTGTACAAACTTGGcattttcactgaaaatg	attB1- *wee-1.3* promoter (reverse)
oAKA5	ggggacagctttcttgtacaaagtggTTATGGACGACACAGAGGGTAACTC	attB2- *wee-1.3* gene (forward)
oAKA6	ggggacagctttcttgtacaaagtggGGTAAtaatgcacaaaaatcagaaa	attB2- *wee-1.3* 3'UTR (forward)
oAKA9	ggggacaactttgtataataaagttgCgtcctacctccagtactacaagaacagg	attB3- *wee-1.3* 3'UTR (reverse)
oAKA51	GGGGACAACTTTGTATAGAAAAGTTGGCaccccaaccattgcaaatatgtg	attB4- *wee-1.3* promoter (forward)
oAKA52	GGGGACTGCTTTTTTGTACAAACTTGCaacctcgtcgcctgatgatcc	attB1- *wee-1.3* gene (reverse)
B4F2	ggggacaactttgtatagaaaagttggcACGTGGATGAATGAGAGCAGC	attB4- *cbd-1* promoter (forward)
B1R3	ggggactgcttttttgtacaaacttggATATCCCGATTTATCATCATTGTACG	attB1- *cbd-1* gene (reverse)
B2rF2	ggggacagctttcttgtacaaagtggccTGATAAGTTGTTCTAAACACCCCC	attB2- *cbd-1* 3'UTR (forward)
B3R1	ggggacaactttgtataataaagttggGCGTCCCTTTCACATTTACAG	attB3- *cbd-1* 3'UTR (reverse)
oAKA70	ggtggttcctccggaaagaa	*act-1* (forward)
oAKA71	gctatgttccagccatccttct	*act-1* (reverse)
oAKA88	gaataatgtgatcgacgaggctcc	*wee-1.3* (forward)
oAKA89	tcttctacgtggcgattccgattg	*wee-1.3* (reverse)
oAKA80	tctcaccatcgttctctctcactg	*vet-1* (forward)
oAKA81	tcggtgggagtgaagaatgg	*vet-1* (reverse)
oAKA84	gtgcttaactttccatccgttg	*vet-4* (forward)
oAKA85	caacccagtttctctggaagtc	*vet-4* (reverse)
oAKA76	ggaagaatgttcaagcggcac	*vet-6* (forward)
oAKA77	tccgtctctgatggcttcttctc	*vet-6* (reverse)
